# A Mutant Variant of E2F4 Triggers Multifactorial Therapeutic Effects in 5xFAD Mice

**DOI:** 10.1007/s12035-022-02764-z

**Published:** 2022-03-07

**Authors:** Noelia López-Sánchez, Morgan Ramón-Landreau, Cristina Trujillo, Alberto Garrido-García, José M. Frade

**Affiliations:** Department of Molecular, Cellular and Developmental Neurobiology, Cajal Institute, Consejo Superior de Investigaciones Científicas, E-28002 Madrid, Spain

**Keywords:** Alzheimer’s disease, E2F4 phosphorylation, RNA-seq, Aβ deposits, Neuronal tetraploidy, Y-maze

## Abstract

Alzheimer’s disease (AD) has a complex etiology, which requires a multifactorial approach for an efficient treatment. We have focused on E2 factor 4 (E2F4), a transcription factor that regulates cell quiescence and tissue homeostasis, controls gene networks affected in AD, and is upregulated in the brains of Alzheimer’s patients and of APP^swe^/PS1^dE9^ and 5xFAD transgenic mice. E2F4 contains an evolutionarily conserved Thr-motif that, when phosphorylated, modulates its activity, thus constituting a potential target for intervention. In this study, we generated a knock-in mouse strain with neuronal expression of a mouse E2F4 variant lacking this Thr-motif (E2F4DN), which was mated with 5xFAD mice. Here, we show that neuronal expression of E2F4DN in 5xFAD mice potentiates a transcriptional program consistent with the attenuation of the immune response and brain homeostasis. This correlates with reduced microgliosis and astrogliosis, modulation of amyloid-β peptide proteostasis, and blocking of neuronal tetraploidization. Moreover, E2F4DN prevents cognitive impairment and body weight loss, a known somatic alteration associated with AD. We also show that our finding is significant for AD, since E2F4 is expressed in cortical neurons from Alzheimer patients in association with Thr-specific phosphorylation, as evidenced by an anti-E2F4/anti-phosphoThr proximity ligation assay. We propose E2F4DN-based gene therapy as a promising multifactorial approach against AD.

**Supplementary Information:**

The online version contains supplementary material available at 10.1007/s12035-022-02764-z.

## Introduction

Alzheimer’s disease (AD) is characterized by progressive neurodegeneration that leads to cognitive impairment and eventually to dementia, in association with somatic alterations that include body weight loss [1]. Two main neuropathological hallmarks, derived from altered proteostasis, can be found in the brain of AD patients, namely, senile plaques containing amyloid-β peptide (Aβ), which is derived from Aβ precursor protein (APP) processing, and intraneuronally located neurofibrillary tangles (NFTs) of hyperphosphorylated tau protein [2]. Compelling evidence indicates that AD has a multifactorial etiology [3], with neuroinflammation as a relevant central mechanism [4], due to its capacity to exacerbate Aβ and tau pathologies [5]. Other early-onset processes that cooperate in the etiology of AD include synapse loss [6], altered glucose metabolism [7], oxidative stress [8], chronic hypoperfusion [9], and neuronal cell cycle re-entry [10], the latter leading to neuronal tetraploidization (NT) [11]. These processes interact with each other resulting in synergistic effects. For instance, neuronal cell cycle re-entry can induce NFTs, extracellular deposits of Aβ, gliosis, synaptic dysfunction, and delayed neuronal cell death, the combination of which can lead to cognitive deficits [12]. Additionally, oxidative stress affects synaptic activity and triggers abnormal cellular metabolism that in turn may affect the production and accumulation of Aβ and hyperphosphorylated tau [8]. Cell cycle-reentry can also cooperate with altered glucose metabolism in the etiology of AD [13], and synapse dysfunction may also underpin AD etiology [14]. The mutual interaction of all of these etiological factors makes it difficult to appropriately target the disease, and no effective therapies against AD are available until now. This is likely due to the monospecific nature of most drugs that have been tested so far. Therefore, a paradigm shift is necessary, making it essential to design a multifactorial approach against this complex disease [3].

A potential multifactorial target for AD is E2 factor 4 (E2F4), a transcription factor proposed as a major regulator of most AD-specific gene networks [15]. Other evidence supporting an active role of E2F4 in AD comes from both bioinformatics-based studies [16–18] and the existence of E2F transcription factor binding sites in distinct AD-related genes [19, 20]. Moreover, E2F4 can potentially regulate over 7000 genes involved in several AD-affected processes, including its well-known cell cycle regulation function, as well as DNA repair, RNA processing, stress response, apoptosis, ubiquitination, protein transport and targeting, protein folding, and I-κB kinase/NF-κB cascade [21]. Additionally, E2F4 can bind to the promoters of 780 transcription factors, which suggest that E2F4 can indirectly regulate broad classes of genes [21], either through Rb family-dependent or independent mechanisms [22]. Supplementary data from this latter study indicate that E2F4 can physically interact with relevant synaptic regulators (fragile X mental retardation 1, fragile X mental retardation syndrome–related protein (FXR) 1, FXR2, and IQ motif and Sec7 domain-containing protein 2), with proteins crucial for intracellular vesicle trafficking and synaptic vesicle recycling (subunit 2 of biogenesis of lysosomal organelles complex-1 (BLOC-1), BLOC-1-related complex subunit 5, and SNARE-associated protein snapin), and with proteostasis regulators (protein-L-isoaspartate(D-aspartate) O-methyltransferase, Prefoldin (PFDN) 1, and PFDN4). Thus, E2F4 may fulfill AD-relevant functions other than those linked to its DNA-binding activity.

E2F4 is a phosphoprotein [22], and previous studies from our laboratory have shown that E2F4 can be phosphorylated by p38^MAPK^ [23], a major stress kinase that is activated in AD [24]. In the chick, this phosphorylation takes place at the Thr261/Thr263 motif, orthologous of Thr249/Thr251 in mouse E2F4 and Thr248/Thr250 in human E2F4 [23]. Accordingly, a recent study has identified Thr248 (Thr249 in mouse E2F4) as a major phosphorylatable Thr residue in E2F4 [22]. E2F4 expression is upregulated in cortical neurons from APP^swe^/presenilin 1 (PS1)^dE9^ (APP/PS1) mice, a known AD mouse model, in association with phosphoThr immunoreactivity [25]. Remarkably, a similar E2F4 upregulation may also be observed in the AD prefrontal cortex [10] and in human neurons derived from familial AD (FAD) patient-specific hiPSCs [18] as well as in cortical neurons from 5xFAD mice [25], another AD mouse model that expresses human APP and PS1 containing five pathological mutations [26].

We have demonstrated that a phosphomimetic form of chick E2F4 with Thr261Glu/Thr263Glu mutations leads to cell cycle re-entry in differentiating chick neurons that lack p38^MAPK^ activity, while a dominant negative form of chick E2F4 (E2F4DN) containing Thr261Ala/Thr263Ala substitutions blocks NGF-induced cell cycle re-entry in these cells [23]. This indicates that phosphorylation by p38^MAPK^ of the Thr conserved motif alters the normal functioning of E2F4 as a quiescent regulator, a process that could participate in the etiology of AD, given that p38^MAPK^ is activated in AD-affected neurons [24]. Therefore, the presence of E2F4DN in p38^MAPK^ expressing cells is expected to conserve E2F4 functionality. The p38^MAPK^-dependent phosphorylation of E2F4 may also alter other homeostatic processes regulated by this transcription factor, and if this is the case, then, E2F4DN may well be a potential therapeutic tool for AD.

In this study, we generated a knock-in mouse strain expressing mouse E2F4DN in neurons, which were mated to 5xFAD mice. We show that neuron-specific expression of E2F4DN in 5xFAD mice prevented NT and induced a transcriptional program that includes markers of synapse formation, improved glucose metabolism and vascular integrity, and decreased oxidative stress, glycophagy, and cell starvation. This program is also compatible with the attenuation of the immune response and of Aβ processing, accumulation, and toxicity. Consistently, both microgliosis and astrogliosis were reduced in 5xFAD/E2F4DN mice. Moreover, although the attenuation of the neuroinflammatory response initially correlated with larger Aβ deposits, Aβ deposition was lessened at later stages, and cognition was preserved in 5xFAD/E2F4DN mice, as is the case in asymptomatic AD individuals [27]. Furthermore, neuronal E2F4DN prevented AD-associated somatic alterations such as body weight loss. We also show that E2F4 can be detected in cortical neurons from Alzheimer patients, associated with Thr-specific phosphorylation. Therefore, we propose E2F4DN as a promising multifactorial therapeutic agent against AD [28].

## Materials and Methods

### Human Cryosections

Cryosections of the parietal cortex from AD patients were provided by the Banco de Tejidos Fundación CIEN (BT-CIEN) (Madrid, Spain). These cryosections were obtained from the right half of the brain, which was cut in slices and frozen in −60 °C isopentane immediately after post-mortem brain extraction. A full neuropathological examination of each brain was conducted on the left half of the brain. The severity of the Alzheimer pathology was scored according to the “National Institute on Aging-Alzheimer’s association guidelines for the neuropathologic assessment of Alzheimer’s disease,” following the “ABC” protocol [29]. Consequently, total amyloid burden (“A” score) was determined according to the Thal staging system, the stage of neurofibrillary pathology was established according to the Braak (“B” score) scheme, and the frequency of neuritic plaques in the associative cortex according to the CERAD protocol (“C” score) was registered. Written informed consent for brain removal after death for diagnostic and research purposes was obtained from the brain donors and/or next of kin. The procedures have been approved by the BT-CIEN Scientific Committee and the Bioethics Committee of the *Consejo Superior de Investigaciones Científicas* (CSIC).

### Mice

**Fig. 1:**
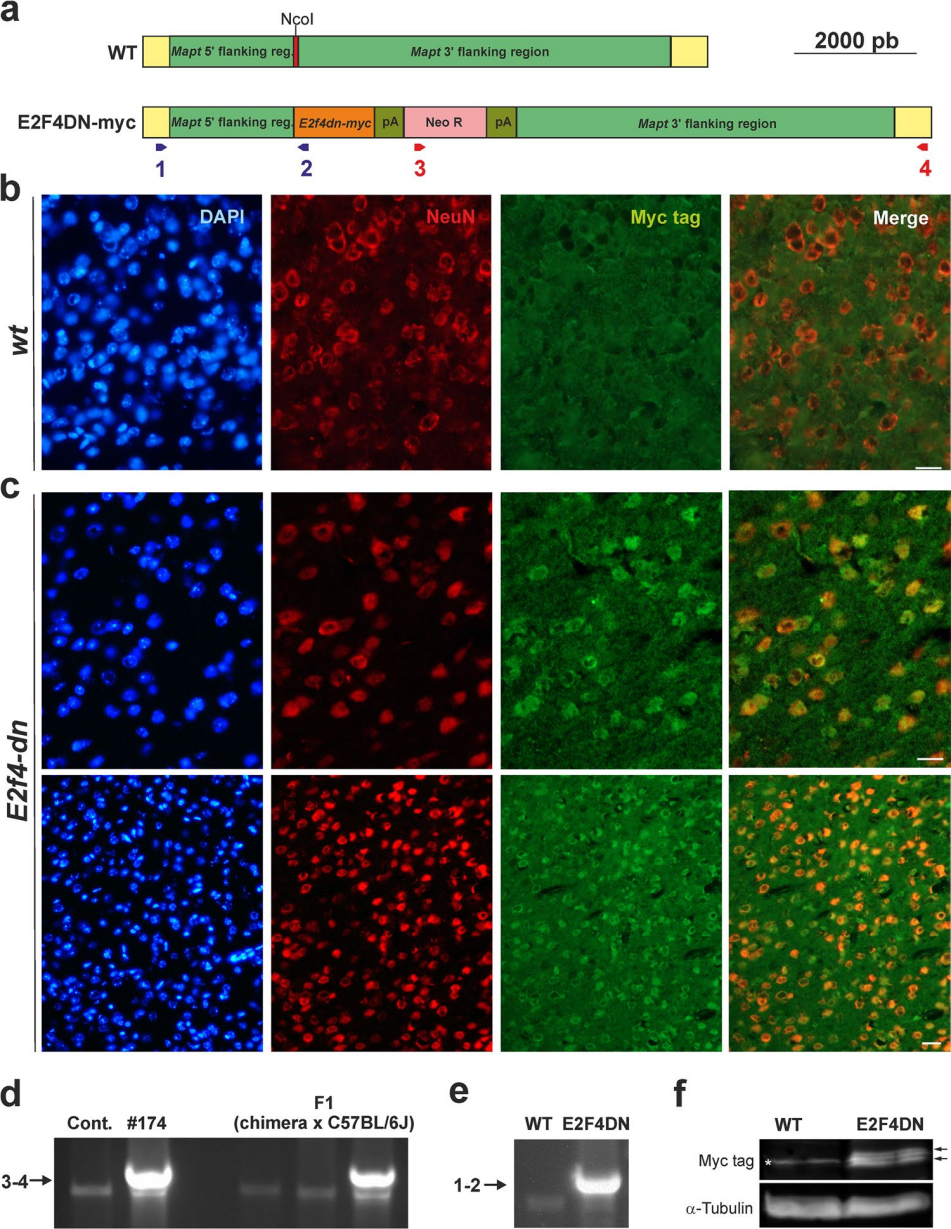
Generation and characterization of E2F4DN mice. **a** Scheme showing the DNA construct used to generate the E2F4DN mice. Red: Mapt Exon I containing the NcoI restriction site where the E2f4dn-myc cassette was inserted. pA: Pgk-1 polyadenylation signal, Neo R: Pgk-NeoR sequence. Primers #1 and #2, for the 5′ flanking region, are shown in blue while primers #3 and #4, for the 3′ flanking region, are depicted in red (see Methods for details). **b** A cerebral cortex cryosection from WT mice immunostained with anti-NeuN (red) and anti-Myc tag (green), and counterstained with DAPI. **c** Cerebral cortex cryosections from E2F4DN mice immunostained with anti-NeuN (red) and anti-Myc tag (green), and counterstained with DAPI. **d** Genomic DNA from control R1 cells (Contr.), clone #174, and three mice descendants from a cross between the founding chimera and a C57BL/6J mouse, amplified with primers #3 and #4. Arrow: band specific for the E2F4DN construct. **e** Genomic DNA from a WT and an E2F4DN mice, amplified with primers #1 and #2. Arrow: band specific for the E2F4DN construct. **f** Western blot performed with extracts from cerebral cortex of WT and E2F4DN mice using an antibody against c-Myc tag. Loading control performed with an anti α-tubulin. Asterisk: unspecific band. Arrows: specific bands. Scale bar: 20 μm (b, c upper panels), 80 μm (c bottom panels)

Experimental procedures with mice were approved by the CSIC animal ethics committee and the Autonomous Government of Madrid, in compliance with Spanish and European Union guidelines. Double transgenic mice in C57BL/6J genetic background expressing under the control of the Thy1 promoter both mutant human APP695 with the Swedish (K670N, M671L), Florida (I716V), and London (V717I) FAD mutations, and human PS1 harboring the M146L and L286V FAD mutations (Tg6799 or 5xFAD mice) [26] were purchased from The Jackson Laboratory (strain #008730). 5xFAD mice were genotyped as indicated by The Jackson Laboratory. A 5xFAD mouse strain was obtained after repeated inbreeding of originally hemizygous mice. Homozygous *Mapt*^*tm1(EGFP)Klt*^ knock-in mice expressing enhanced green fluorescent protein (EGFP) in neurons (EGFP mice) [30] were purchased from The Jackson Laboratory (strain #004779). EGFP mice have a target mutation in the *Mapt* gene, in which the coding sequence of EGFP has been inserted into the first exon, thus disrupting the expression of the tau protein. This results in the neuron-specific expression of cytoplasmic EGFP. Tau is expressed at high levels in neurons [31], and homozygous mice mutant for tau are viable, fertile, and display no gross morphological abnormalities in the central or peripheral nervous systems [30]. Homozygous EGFP mice are viable, fertile, normal in size, and do not display any gross physical or behavioral abnormalities. EGFP mice were genotyped as indicated by The Jackson Laboratory. These mice were used in this study as a control for E2F4DN mice. Homozygous EGFP mice were bred with hemizygous 5xFAD mice to generate littermates consisting of hemizygous EGFP mice with or without the 5xFAD transgene. *Mapt*^*tm(mE2F4DN-myc)*^ knock-in mice (E2F4DN mice) were generated following the procedure described by [30]. These mice express a dominant negative form of E2F4 equivalent to the mutant E2F4 used to prevent NT in chick neurons [23]. To this end, a cassette containing the coding sequence of mouse E2F4 with the Thr249Ala/Thr251Ala mutations followed by the c-Myc tag, the Pgk-1 polyadenylation signal, and the G418-selectable marker Pgk-NeoR was inserted into the NcoI site of a plasmid containing exon 1 of the Mapt gene and 8.0 kb flanking genomic sequence. The linearized targeting vector was electroporated into 129Sv-derived R1 embryonic stem cells. One hundred eighty-two G418-resistant colonies were analyzed by genomic PCR using Taq DNA polymerase (BioTools) to verify the upstream inserted sequence using primers #1 and #2 (5′AGGAGGCAGAAACAAGTGGA3′ and 5′ACACGAACTTGGTGGTGAGA3′, respectively; amplicon: 2,160 bp) (Fig. 1a). Nine of the analyzed clones produced the expected band. These clones were further analyzed by genomic PCR using Long Amp Taq DNA polymerase (New England Biolabs) to check the downstream inserted sequence. For this analysis, the following primers were used: 5′GGCGCCCGGTTCTTTTTGTC3′ (primer #3) and 5′CACACAGCTAGTCCACAAAG3′ (primer #4) (amplicon 7951 bp) (Fig. 1a). Two clones (#138 and #174) were found to produce the expected band. After confirming the euploidy of the latter clone, it was used to make chimeras by injecting into C57BL/6 blastocysts, and four male high-percentage chimeras were mated to C57BL/6 females. Genomic PCR using the oligonucleotides that amplify the upstream sequence (primers #1 and #2) indicated that agouti offspring of chimeric males (*n* = 25) carried the transgene at the expected Mendelian frequency. The subsequent progeny was analyzed by genomic PCR with primers #1 and #2 (E2F4DN band) and the wild-type (WT) primers described by The Jackson Laboratory for EGFP mice (5′-CTCAGCATCCCACCTGTAAC-3′ and 5′-CCAGTTGTGTATGTCCACCC-3′). The knock-in strain was maintained on a mixed background of C57BL/6 and 129Sv or backcrossed to the C57BL/6 background. Homozygous E2F4DN mice were created by inbreeding mice containing one copy of the E2F4DN transgene. Homozygous E2F4DN mice are viable, fertile, normal in size, and do not display any gross physical or behavioral abnormalities, even though the tau protein has been deleted [30]. Homozygous E2F4DN mice were bred with hemizygous 5xFAD mice to generate littermates consisting of hemizygous E2F4DN mice with or without the 5xFAD transgene. Analyses were performed in hemizygous mice for both *Egfp* and *E2f4dn* transgenes to avoid the observed effects of full Mapt null mutation in the phenotype of APP and APP/PS1 transgenic mice [32]. E2F4DN mice are available upon request for research purposes other than neurological and neurodegenerative diseases.

### Antibodies

Rabbit anti-Myc tag polyclonal antibody (pAb) (ab9106; Abcam) was used at 1:1000 for western blotting and at 1:500 for immunohistochemistry. Mouse anti-α-tubulin monoclonal antibody (mAb) clone DM1A (ab7291; Abcam) was used at 1:10,000 for western blotting. The rabbit anti-NeuN pAb (ABN78, Merck Millipore) was diluted to 1:800 for flow cytometry and 1:1000 for immunohistochemistry. The mouse anti-NeuN mAb, clone A60 (MAB377; Merck Millipore) was used at 1:1600 dilution for immunohistochemistry. The rabbit anti-GFAP pAb (ab7260, Abcam) was diluted to 1:1000 for immunohistochemistry. The rabbit anti-Iba1 pAb (019-19741, Wako) was used at 1:800 dilution for immunohistochemistry. The rabbit anti-E2F4 polyclonal antibody (LS-B1532; LSBio) was used at 1:400 for proximity ligation assay (PLA) and immunohistochemistry. Mouse anti-E2F4 mAb clone LLF4-2 (MABE160; Merck Millipore) used at 1:400 for PLA and 1:6,000 for western blot. Mouse anti-phosphoThr mAb clone 20H6.1 (05-1923; Merck Millipore) was used at 1:750 for PLA. The chicken anti-NeuN pAb (AP31812PU-N, Acris) was diluted 1:500 for PLA. The mouse anti-Synaptophysin mAb SY38 (PROGEN) was diluted 1:500 for western blot. The mouse anti-Actin monoclonal antibody (mAb), clone C4 (MAB1501R; Merck Millipore) was used at 1:10,000 for western blotting. The rabbit anti-β-Amyloid pAb #2454 (Cell Signaling Technology) was diluted 1:1,000 for western blotting. The APP mAb 22C11 (Invitrogen) was used at a dilution of 1:3,000 for western blotting. The anti-Tau pAb ab64193 (abcam) was diluted 1:1000 for western blotting.

The donkey anti-rabbit IgG (H + L) highly cross-adsorbed secondary antibody, Alexa Fluor 488 (Invitrogen) was used at 1:1000 dilution for immunohistochemistry and 1:400 for flow cytometry. The goat anti-mouse IgG (H + L) cross-adsorbed secondary antibody, Alexa Fluor 568 (Invitrogen) was diluted 1:1,000 for inmunohistochemistry. The goat anti-chicken IgY (H + L) secondary antibody, Alexa Fluor 647 (Invitrogen) was used at 1:800 dilution for PLA. The IRDye 800CW Goat anti-Rabbit IgG (H + L) and IRDye 680RD Goat anti-Mouse IgG (H + L) antibodies (LI-COR) were diluted 1:15,000 for western blotting.

### RNA Extraction and cDNA Synthesis

Total RNA was extracted using QIAzol Reagent (Qiagen), and cDNA was synthesized using SuperScript IV Reverse Transcriptase (ThermoFisher Scientific) following the manufacturer’s instructions.

### RNA Extraction and RNA-Seq Library Preparation

Total RNA was extracted from the cerebral cortex of two 5xFAD/EGFP and two 5xFAD/E2F4DN female mice using TriZOL (ThermoFisher Scientific) according to the recommended protocol. Residual genomic DNA was removed with DNase I recombinant, RNase-free (Roche), following the manufacturer’s instructions. The concentration and quality of the total RNAs were measured using an Agilent Bioanalyzer 2100 with RNA 6000 nano Chips (Agilent Technologies). All samples had an RNA integrity value of 7 or greater. 1000 ng of RNA from the combination of two mouse brains of each genotype were used for each RNA-Seq library, which was created using the “NEBNext Ultra Directional RNA Library preparation kit for Illumina” (New England Biolabs) following the manufacturer’s instructions. We followed the indications of “Chapter 1: Protocol for use with NEBNext Poly(A) mRNA Magnetic Isolation Module.” We performed the library amplification included in the manufacturer’s instructions using a 12-cycle PCR. The quality of the RNA-Seq libraries was assessed with an Agilent Bioanalyzer and Agilent DNA7500 DNA chips to confirm that the insert sizes were 200–400 bp (average size: 299-317) for all individual libraries. The individual libraries obtained were also quantified by an Agilent 2100 Bioanalyzer with a DNA7500 LabChip kit and an equimolecular pool of libraries were titrated by quantitative PCR using the “Kapa-SYBR FAST qPCR kit for LightCycler 480” (Kapa BioSystems) and a reference standard for quantification. The pool of libraries was denatured prior to seeding on a flowcell at a density of 2.2 pM, where clusters were formed and sequenced using a “NextSeq™ 500 High Output Kit” (Illumina Inc.).

### RNA Sequencing and Bioinformatic Analysis of RNA-Seq Data

RNA-Seq libraries were single-end sequenced in a 1 × 75 format using an Illumina NextSeq500 sequencer at the Genomic Unit of the Scientific Park of Madrid, Spain. The raw reads (FD: 34,860,735 reads, FT: 29,591,070 reads) passed the quality analysis conducted with the FastQC tool. Trimmed reads were subsequently mapped to the Genome Reference Consortium Mouse Buildt 38 patch release 5 (GRCm38.p5), adding the EGFP sequence with the Bowtie tool [33] included in the TopHat suite [34]. The resulting alignment files were used to generate a transcriptome assembly for each condition with the Cufflinks tool, and the expression levels were then calculated with the Cuffdiff tool, together with the statistical significance of each observed change in expression [34]. *p* < 0.05 was used as a criterion for differential expression since the FAD/EGFP vs FAD/E2F4DN comparison yielded no gene tagged as significantly expressed by Cufflinks. As described in the main text, differential expression in several genes was confirmed by qPCR.

### qPCR

Reverse transcriptase (RT)-qPCR was performed with the 7500 real-time PCR equipment (Applied Biosystems), using specific primers from PrimePCR SYBR Green Assay (BioRad) and the house keeping gene *Rps18* (qMmuCED0045430 PrimePCR SYBR Green Assay; BioRad). ΔCt for treatment and control was calculated, and subsequently, the statistical significance was evaluated by a post hoc Student’s *t* test in genes where two-way ANOVA analysis was found to be significant. No statistically significant changes were observed among the average Ct values of *Rps18* for the different genotypes (WT/EGFP: 20.87 ± 0.41, WT/E2F4DN: 21.03 ± 0.61, 5xFAD/EGFP: 20.95 ± 0.46, 20.90 ± 0.43 (mean ± SEM); *n* = 3; Student’s *t*-test).

### Tissue Processing

After anesthetizing the mice with intraperitoneal sodium pentobarbital (Dolethal; Vetoquinol), administered at 50 mg/kg (body weight), they were transcardially perfused with PBS, and then with 4% paraformaldehyde (PFA). Brains were finally postfixed overnight at 4 °C with 4% PFA and cryoprotected by sinking in 30% sucrose in PBS at 4°C. The brains were then embedded in either Tissue-Tek (Sakura) followed by freezing in dry ice to get cryosections (12–15 μm), or in 3% agarose gels prepared in 0.1 phosphate buffer, pH 7.37, before cutting them with a vibratome (50 μm).

### Thioflavin S Staining

The vibratome sections were washed three times with phosphate-buffered saline (PBS) containing 0.4% Triton X-100 (Sigma-Aldrich) (0.4% PBTx), and then incubated for 30 min in the dark with 0.05% Thioflavin S (Sigma-Aldrich) in 50% ethanol (Merck). Finally, the sections were washed twice with 50% ethanol, and once with distilled water. Then, the sections were subjected to immunohistochemistry as described below.

### Immunohistochemistry

The cryosections were permeabilized and blocked for 1 h at RT in 0.1% PBTx and 10% fetal calf serum (FCS; Invitrogen), and incubated overnight (O/N) at 4 °C with the primary antibodies in 0.1% PBTx plus 1% FCS. After washing with 0.1% PBTx, the sections were incubated for 1 h at RT in 0.1% PBTx plus 1% FCS with the secondary antibodies. The sections were washed in 0.1% PBTx, and then incubated with 100 ng/ml 4′,6-diamidine-2′-phenylindole dihydrochloride (DAPI; Sigma-Aldrich) in PBS before mounting with ImmunoSelect antifading mounting medium (Dianova). The vibratome sections were permeabilized and blocked in 0.4% PBTx containing 10% FCS for 3 h. They were then incubated overnight at 4 °C with the primary antibodies in 0.1% PBTx containing 1% FCS. After five washes of 20 min with 0.1% PBTx, the sections were incubated with the secondary antibodies plus 100 ng/ml DAPI in 0.1% PBTx for 3 h at room temperature (RT). The sections were then washed five times with 0.1% PBTx, and mounted with ImmunoSelect antifading mounting medium.

### Quenching of Lipofuscin Autofluorescence Signal

Lipofuscin present in the brains of 6-month-old mice was quenched with TrueBlackTM Lipofuscin Autofluorescence Quencher (Biotium). Briefly, the vibratome sections were washed once with PBS and treated for 30 s with TrueBlack 1x prepared in 70% ethanol. Finally, the sections were washed three times with PBS, and then immunostained as described above.

### PLA

PLA was performed in cryostat sections (4–6 μm) obtained from frozen samples of human parietal cortex (one Braak I patient and one Braak VI patient) using the Duolink In Situ Detection Reagents Brightfield system (Sigma-Aldrich). This method monitors the coincidence of specific epitopes and has been previously used for monitoring protein phosphorylation [35]. The cryosections were blocked for 90 min at RT with Tris-buffered saline (TBS) containing 0.2% Triton X-100 (Sigma-Aldrich) and 10% bovine serum (Invitrogen), and then, endogenous peroxidase was quenched for 30 min at RT with 3% hydrogen peroxide in TBS. The cryosections were then incubated O/N at 4 °C with the primary antibodies (chicken anti-NeuN, Rabbit anti-E2F4, and either mouse anti-E2F4 or mouse anti-phosphoThr), diluted in TBS containing 0.1% Triton X-100 (TBTx) plus 1% bovine serum. After washing with TBTx, the cryosections were transferred to TBS and incubated for 1 h at 37 °C with anti-mouse PLUS and anti-Rabbit MINUS PLA probes (Sigma-Aldrich). The cryosections were then incubated for 30 min at 37 °C with 1× ligation mixture, for 135 min at 37 °C with the amplification solution, for 60 min at RT with the detection solution, and for 10 min at RT with the substrate solution, following the manufacturer’s instructions. Finally, the cryosections were washed twice with TBS and incubated for 1 h at RT with the secondary anti-chicken antibody in TBTx containing 100 ng/ml DAPI. The cryosections were then washed with TBTx and mounted in PBS/Glycerol (1:1).

### Confocal Microscopy and Image Analysis

Confocal images were acquired at 20× magnification with a Leica SP5 confocal microscope. Image analysis was performed using ImageJ (Fiji). The images used for the analysis (at least two mosaic images per tissue and animal) were maximum intensity projections, created as output images whose pixels correspond to the maximum value of each pixel position (in *xy*) across all stack images (*z*). In order to analyze the area occupied by GFAP and Iba1, a threshold was set to highlight the area to be quantified. In order to analyze the number and size of Aβ deposits, the region of interest (ROI) tool was used to delimitate the different cortical layers, and a threshold was then set to highlight the area to be quantified. An average of 100 plaques per individual were analyzed. The MorphoLibJ (v1.3.1) integrated library (https://imagej.net/plugins/morpholibj) and plugin was used to analize the soma size and roundness (R), as previously described [36]. Quantification of the area occupied by Iba1 labeled microglia was achieved using a multi-step algorithm. First, Iba1 labeled microglia were segmented by applying a grey scale attribute opening filter (area minimum: 25 pixels; connectivity: 8) to an 8-bit maximum projection. An opening morphological filter (1-pixel radius octagon) was then used effectively to separate microglia soma from processes, before a maximum entropy threshold was used to segment microglia soma from the image background. The quantification of microglia soma and R was completed using the Fiji (ImageJ) Analyse Particles function, with a particle size threshold of 10 pixels to exclude small pixel noise. The algorithm used to calculate *R* was $R=\frac{4A}{\pi {M}^2}$, where *A* is the area of the microglia soma and *M* is the length of the major axis, derived from the longest axis of an ellipse fit to each microglia soma.

### Western Blotting

Cortical and hippocampal extracts were obtained in cold extraction buffer (20 mM Tris-HCl pH 6.8, 10 mM β-mercaptoehanol (Sigma-Aldrich), 1 mM EDTA (Merck), 1% Triton X-100, 1% SDS (Sigma-Aldrich)) including 1x cOmplete Mini, EDTA-free, protease inhibitor cocktail (Roche) (one hemicortex in 500 μl extraction buffer). The extracts were centrifuged for 10 min at 14,000 ×g (at 4 °C), and supernatants were then boiled for 5 min in Laemli buffer. The extracts were fractionated by SDS PAGE on 10% acrylamide gels and transferred to Immobilon-FL membranes (Millipore). The membranes were incubated for 1 h with Odyssey Blocking Buffer TBS (LI-COR) (OBB), and then incubated ON at 4 °C with the appropriate antibody in OBB containing 0.1% Tween 20. For Aβ analysis, hippocampal extracts prepared as described above were boiled for 5 min in 50 mM Tris-HCl pH 8.0 containing 12% Glicerol (Merck), 4% SDS, 0,01% Coomasie Brilliant Blue G-250 (Sigma-Aldrich), and 2% β-mercaptoethanol. Extracts were fractionated on 16.5% Mini-PROTEAN Tris-Tricine gels (Bio-Rad) (30 V for the first hour and then 125 V) using 1x Tris/Tricine/SDS Running Buffer (Bio-Rad) (cathode) and 200 mM Tris-HCl pH 9.0 (anode), and then transferred to Pierce Low-Fluorescence PVDF Transfer Membranes (0.2 μm) (ThermoFisher Scientific). The membranes were incubated for 1 h with Intercept Blocking Buffer TBS (LI-COR) (IBB), and then incubated ON at 4 °C with the rabbit anti-β-Amyloid pAb (1:1000) in IBB containing 0.1% Tween 20. After washing the membranes five times in TBS containing 0.1% Tween 20 (TBS-T), they were incubated for 1 h at RT with a 1:15,000 dilution of secondary antibodies in OBB (IBB for Aβ analysis) containing 0.1% Tween 20. Finally, they were washed again with TBS-T as described above, and the protein bands were visualized using the Odyssey CLx Infrared Imaging System (LI-COR).

### Cell Nuclei Isolation

Cell nuclei isolation was performed as described by [37]. Briefly, fresh-frozen mouse cerebral hemicortices were placed in 2.5 ml ice-cold, DNase-free 0.1% PBTx and protease inhibitor cocktail (Roche) (nuclear isolation buffer). Cell nuclei were then isolated by mechanical disaggregation using a dounce homogenizer. Undissociated tissue was removed by centrifugation at 200×g for 1.5 min at 4 °C. The supernatant was 8-fold diluted with nuclear isolation buffer and centrifuged at 400×g for 4 min at 4 °C. Supernatant with cellular debris was discarded, and the pellet incubated at 4 °C in 800–1000 μl cold nuclear isolation buffer for at least 1 h, prior to mechanical disaggregation by gently swirling the vial. The quality and purity of the isolated nuclei were analyzed microscopically after staining with 100 ng/ml DAPI.

### Flow Cytometry

Flow cytometry was conducted as described by [37]. Cell nuclei were immunostained by adding both primary (rabbit anti-NeuN) and secondary (Alexa 448-coupled anti-Rabbit) antibodies to 400 μl of isolated unfixed nuclei containing 5% of FCS and 1.25 mg/ml of BSA. In control samples, the primary antibody was excluded. Finally, the reaction was incubated O/N at 4 °C in the dark. Immunostained nuclei (400 μl) were filtered through a 40-μm nylon filter, and the volume adjusted to 800–1000 μl with DNase-free 0.1% PBTx containing propidium iodide (PI; Sigma-Aldrich) and DNAse-free RNAse I (Sigma-Aldrich) at a final concentration of 40 μg/ml and 25 μg/ml, respectively, and incubated for 30 min at RT. The quality of the nuclei and specificity of the immunostaining signal was checked with fluorescence microscopy. Flow cytometry was then conducted with a FACSAria I cytometer (BD Biosciences, San Diego, CA) equipped with a 488-nm Coherent Sapphire solid state and 633-nm JDS Uniphase HeNe air-cooled laser. Data were collected by using a linear digital signal process. The emission filters used were BP 530/30 for Alexa 488, and BP 616/23 for PI. The data were analyzed with FACSDiva (BD Biosciences). Electronic compensation for fluorochrome spectral overlap during multi-color immunofluorescence analysis was carried out when needed. Cellular debris, which was clearly differentiated from nuclei due to its inability to incorporate PI, was gated and excluded from the analysis. DNA content histograms were generated excluding doublets and clumps by gating on the DNA pulse area versus its corresponding pulse height. The exclusion of doublets was confirmed by checking the DNA pulse area versus the pulse width of the selected population, and the percentage of tetraploid nuclei was quantified. A minimum of 15,000 and 20,000 nuclei were analyzed for the NeuN-positive population. The proportion of tetraploid nuclei was normalized to the value obtained in cell nuclei from 2-month-old control WT mice as shown by [38], which was used as an internal control in all the experiments.

### Activity Cage Test

Exploratory locomotor activity was recorded using a VersaMax Animal Activity Monitoring System (AccuScan Instruments, Inc.) in an open field (40 cm × 40 cm) over a 10-min period. Infrared beams automatically record horizontal movements and rearing in the open field. The task analyzes the activity behavior by measuring the number of beams that are broken during the designated period of time. Ten trials repeated within two consecutive days (five trials/day) were performed for every animal and the results were expressed as average number of broken beams per trial.

### Rotarod Test

Motor coordination was evaluated in a Rotarod apparatus (Ugo Basile) with increasing acceleration. The apparatus consisted of a horizontal motor-driven rotating rod in which the animals were placed perpendicular to the long axis of the rod, with the head placed against the direction of rotation so that the mouse must move ahead in order to avoid falling. The trial was stopped when the animal fell down or after a maximum of 5 min. The time spent in the rotating rod was recorded for each animal and trial. Animals received a pretraining session to familiarize them with the procedure prior to evaluation. Thereafter, a total of six consecutive trials were conducted done for each animal. The data are presented as the average time spent before falling from the apparatus.

### Spontaneous Alternation Y-Maze Test

Each mouse was placed within the center of a Y-maze apparatus (Panlab) and then allowed to freely explore the different arms during an 8-min session. The sequence of arms entered was recorded, and working memory was measured as the percentage of alternation (p.a.), which was calculated as the number of triads containing entries in all three arms divided by all the triads and then multiplied by 100.

#### MWM

The MWM test was used to evaluate spatial learning. The apparatus was a circular tank 100 cm in diameter, filled with water (at 21–22 °C) made opaque. A platform was hidden inside the tank 2 cm below the water level. The experiment consisted of five sessions of four trials 30–60 min apart, performed under constant illumination conditions (7–15 lux). Each of the four starting positions (N, S, E, W) was used randomly in every daily session. Each trial was terminated when the mouse located the platform or when 60 s had elapsed, followed by a period of 15–20 s in which the animal was allowed to stay on the platform. Several fixed extra maze cues were constantly visible from the pool. All trials were recorded by a video camera located 2 m above the water level. Mice trajectories were analyzed using the Ethovision 3.1 computerized tracking system (Noldus), to measure escape latency for each animal in each trial.

### Bioinformatics Analysis

The DAVID bioinformatics platform was used for Gene Ontology (GO) functional annotation of gene sets (GOTERM_BP_ALL). MGI Mammalian Phenotype (Level 4 2019) was browsed using the Enrichr bioinformatics platform (http://amp.pharm.mssm.edu/Enrichr).

### Statistical Analysis

The quantitative data are represented as the mean ± SEM. Statistical differences in experiments performed with transgenic mice on either EGFP or E2F4DN background were analyzed using two-tailed Student’s *t* test. Two-way ANOVA analysis was performed in qPCR-based experiments, followed by post hoc Student’s *t* test. One-way ANOVA analysis was performed for the quantitative analysis of immune cells, followed by post hoc Newman-Keuls test. Outliers, as evidenced by the Grubbs’ test (transgenic mice experiments) or Dixon’s Q test (qPCR), were eliminated from the analysis.

## Results

### Multifactorial Modulation of Gene Networks by Neuronal E2F4DN Expression in 5xFAD Mice

E2F4 is mainly expressed by cortical neurons (Supplementary Fig. 1). We generated a knock-in mouse strain expressing E2F4DN in neurons (E2F4DN mice). This was accomplished by inserting the coding sequence of mouse E2F4 containing Thr249Ala/Thr251Ala mutations, Myc tagged at the C-terminus, into the gene encoding the microtubule-associated protein tau (*Mapt*), as previously described by Yves-A. Barde’s laboratory for EGFP knock-in mice (EGFP mice) [30] (Fig. 1a). This construct was germ-line transmitted to the progeny (Fig. 1d), as evidenced by primers amplifying the 3′ region of the inserted construct (primers #3 and #4 indicated in Fig. 1a). The insertion of the construct in E2F4DN transgenic mice was confirmed with primers amplifying the 5′ region of the inserted construct (primers #1 and #2 indicated in Fig. 1a, e) and, as expected, neurons from E2F4DN mice expressed the E2F4DN-myc protein, as demonstrated by NeuN/Myc-specific immunohistochemistry (Fig. 1b, c). Western blotting confirmed the specific expression of E2F4DN-myc in the cerebral cortex of these mice (Fig. 1f). As previously reported for EGFP mice [30], the homozygous E2F4DN mice were viable and fertile.

**Fig. 2:**
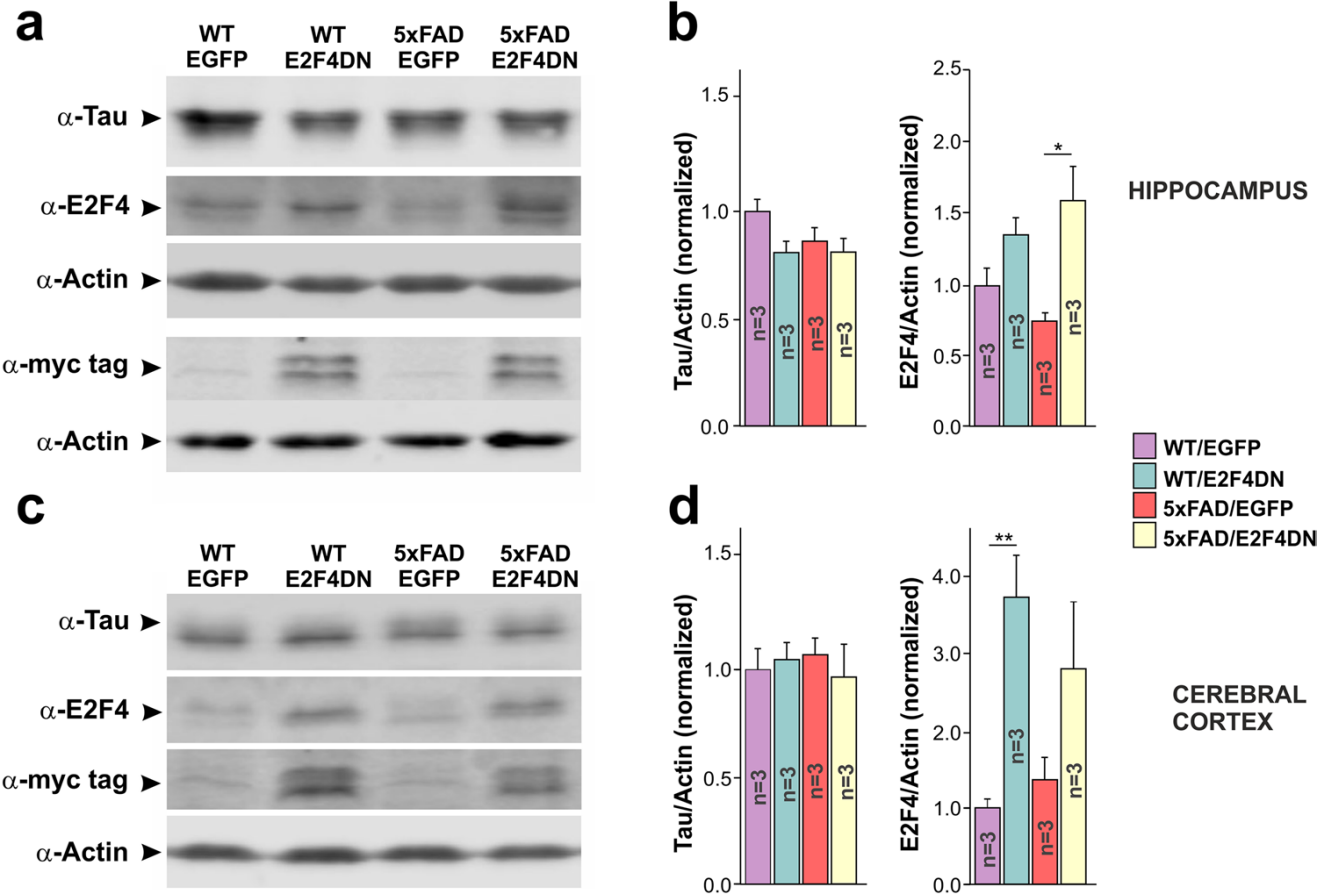
Expression of tau, E2F4 and E2F4DN-myc in the hippocampus and cerebral cortex of transgenic mice. **a** Western blot analysis of hippocampal extracts from 3-month-old mice from the indicated genotypes (*n* = 3 females/genotype, except WT/E2F4DN: 1 male and 2 females) using antibodies against tau protein, E2F4, myc tag and Actin (as a loading control). Original western blots from this and the rest of figures can be seen in Supplementary Fig. 15. **b** Quantification of the ratios of Tau and E2F4 vs Actin in the hippocampus from the indicated genotypes. *p < 0.05 (two-way ANOVA, followed by post hoc Student’s *t* test). **c** Western blot analysis of cortical extracts from 3-month-old mice from the indicated genotypes (*n* = 3 females/genotype, except WT/E2F4DN: 1 male and 2 females) using antibodies against tau protein, E2F4, myc tag, and Actin (as a loading control). **d** Quantification of the ratios of Tau and E2F4 vs Actin in the cerebral cortex from the indicated genotypes. **p < 0.01 (two-way ANOVA, followed by post hoc Student’s *t* test)

To identify transcriptional changes induced by neuronal E2F4DN in AD, we crossed 5xFAD mice with either E2F4DN or control EGFP mice. Western blot analysis demonstrated that E2F4DN was present in both 5xFAD/E2F4DN and WT/E2F4DN mice, while similar levels of tau protein were detected in the four genotypes derived from this cross (Fig. 2). As expected, the presence of the transgene within the *Mapt* locus resulted in reduced expression of tau protein, as compared with mice carrying the WT *Mapt* locus (Supplementary Fig. 2). We then performed RNA-seq analysis using total RNA isolated from the cerebral cortex of 5xFAD/EGFP and 5xFAD/E2F4DN mice of 3 month of age, a stage in which incipient AD-associated neuropathology is already evident [39]. This analysis indicated that in addition to the transgenes, 275 genes were differentially expressed between both genotypes, 71 of which have been documented as participating in AD (Supplementary Table 1). *E2f4* was 2.4-fold enriched in 5xFAD/E2F4DN mice, indicating that the E2F4DN transgene is expressed at physiological levels, a finding consistent with the western blot analysis shown above.

Gene ontology analysis of the differentially expressed genes indicated that the expression of E2F4DN results in a significant change for biological processes related to both immune response and tissue homeostasis, the latter including stress response, positive regulation of metabolism, transcription regulation, MAPK regulation, protein phosphorylation, cell death, and extracellular matrix organization (Supplementary Table 2). The proteins encoded by the differentially expressed genes are likely located at the extracellular space, cell surface (plasma membrane receptor complexes), and at intracellular and extracellular vesicles, including exosomes and lysosomes, as demonstrated by cellular components defined by the GO analysis (Supplementary Table 2). Finally, the molecular functions modulated by E2F4DN are mainly focused on protein binding and transcriptional activity (Supplementary Table 2).

### E2F4DN Expression Modulates Gene Networks Controlling the Immune Response in 5xFAD Mice

**Fig. 3:**
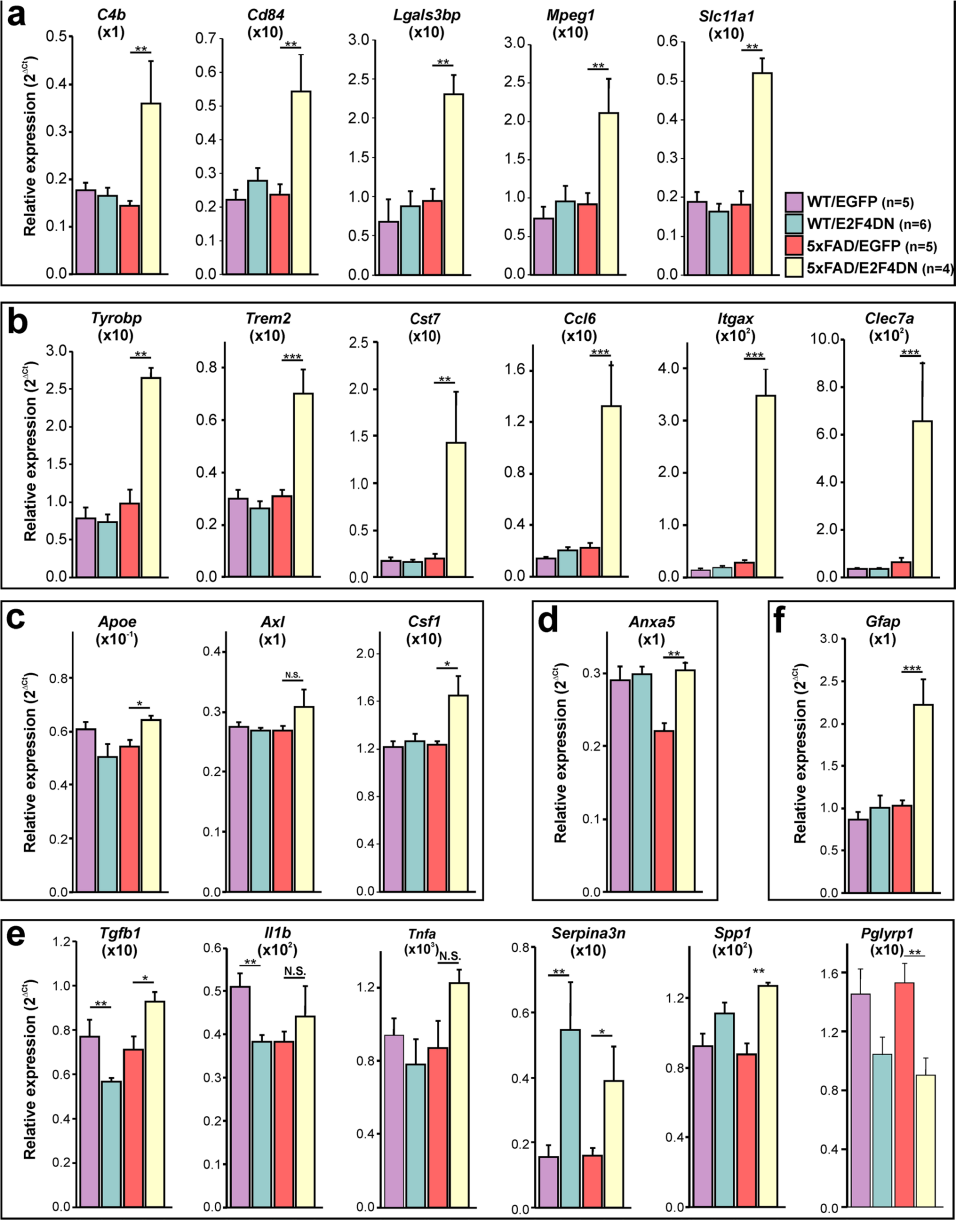
Gene expression in the cerebral cortex of 3-month-old mice of the indicated genotypes. qPCR analysis of genes modulated in the cerebral cortex of WT/EGFP (5 females), WT/E2F4DN (3 males and 3 females), 5xFAD/EGFP (5 females), and 5xFAD/E2F4DN (4 females) mice (**a**), strongly upregulated DAM genes (**b**), low or non-upregulated DAM genes (**c**), phagocytosis-related genes (**d**), inflammation-related genes (**e**), and *Gfap*, an astrocyte marker (**f**). Relative gene expression was normalized to *Rps18* rRNA levels and expressed as 2ΔCt (obtained values were adjusted by the factor indicated between brackets). *p < 0.05; **p < 0.01; ***p < 0.001 (unbalanced two-way ANOVA, followed by post hoc Student’s *t* test)

We compared the differentially expressed genes encoding characterized proteins (248 in total) with those genes whose expression is modulated in the cerebral cortex of APP/PS2 mice [40], another related murine model of Alzheimer. Consistent with the important role played by neuroinflammation in AD [4], Srinivasan et al. [40] found that 84 genes plus an unprocessed pseudogene were upregulated in APP/PS2 mice (74 of microglial origin) (Supplementary Table 3). A total of 36 of these genes were also upregulated in the cerebral cortex of 5xFAD/E2F4DN mice (Supplementary Table 3). We confirmed the upregulation of a selected subset of these genes by qPCR, including *C4b*, *Cd84*, *Lgals3bp*, *Mpeg1*, and *Slc11a1* (Fig. 3a). Functional GO term annotation indicated that the microglia-expressed genes common to 5xFAD/E2F4DN and APP/PS2 mice are mostly involved in innate immune response (Common Genes; Supplementary Table 4). In contrast, the microglia-specific genes unique in APP/PS2 mice (Supplementary Table 3) were mainly involved in the positive regulation of cytokine production (APP_PS2 Unique Genes; Supplementary Table 4). This suggests that neuronal E2F4DN potentiates a non-cytotoxic immune response in the cerebral cortex of 5xFAD mice.

The hypothesis that neuronal expression of E2F4DN favors a non-cytotoxic immune response was further supported by browsing the MGI Mammalian Phenotype database against the protein-encoding genes that are modulated by E2F4DN in the cerebral cortex of 5xFAD mice and are absent in the study by Srinivasan et al. [40] (212 in total). Decreased interferon-gamma secretion and abnormal immune system physiology were the two major phenotypes obtained, while decreased CD4-positive, alpha beta T cell number, decreased interleukin-12 secretion, abnormal T cell activation, and abnormal cytokine secretion were found among other prominent phenotypes (MGI Mammalian Phenotype; Supplementary Table 4).

To further explore the effect of E2F4DN on the immune system, we focused on the specific molecular signature of disease-associated microglia (DAM) [41], recently renamed as activated response microglia [4]. This signature can be modulated to either proinflammatory or anti-inflammatory DAM states [42]. In the cerebral cortex of 5xFAD mice, the transition from homeostatic microglia to the DAM population is a gradual process, initiated by 3–4 months of age [39, 41]. RNA-seq analysis indicated that neuronal E2F4DN accelerated this process in the cerebral cortex of 5xFAD mice, since several DAM-specific genes including the stage 1 DAM genes *Tyrobp*, *Ctsd*, and *Lyz2*, and the stage 2 DAM genes *Trem2*, *Cst7*, *Ccl6*, *Itgax*, *Clec7a*, and *Lilrb4* [41] were largely upregulated by E2F4DN, but not by EGFP (Fig. 3b). In contrast, and consistent with the accelerated expression of immune genes in 5xFAD mouse hippocampus [39], there was a significant increase of microglial markers, including both DAM-specific (Supplementary Fig. 3a) and innate immune response (Supplementary Fig. 3b) genes, in the hippocampus of 3 month-old 5xFAD/EGFP mice. As in the cerebral cortex, neuronal E2F4DN expression largely potentiated the expression of the immune-specific genes in this latter tissue (Supplementary Fig. 3a, b).

Interestingly, in contrast with the strong upregulation of most DAM-specific genes (Fig. 3b), other important DAM genes were only slightly upregulated in the cerebral cortex of 5xFAD/E2F4DN mice (Fig. 3c), suggesting that the DAM phenotype was modulated by neuronal expression of E2F4DN. One of these genes was *Apoe*. Therefore, the capacity of ApoE to prevent the expression of the anti-inflammatory factor TGFβ and to favor cytotoxicity by microglial cells [43] as well as to potentiate the activated response to Aβ [4] seems to be attenuated in the DAM-like phenotype observed in 5xFAD/E2F4DN mice. Accordingly, *Tgfb* was upregulated in the cerebral cortex of 5xFAD/E2F4DN mice (Fig. 3e), while a number of pro-inflammatory DAM cell markers, including *Ptgs2*, *Il1b*, *Il12b*, *Cd44*, *Kcna3*, *Nfkb1*, *Stat1*, and *Rela* [44], were not significantly upregulated in this tissue (Supplementary Table 1). This latter observation was confirmed by qPCR using *Il1b* as a representative pro-inflammatory gene (Fig. 2e). Moreover, *Tnfa*, which encodes the proinflammatory cytokine TNFα, was also not significantly upregulated in the cerebral cortex of 5xFAD/E2F4DN mice (Fig. 3e), providing further evidence that E2F4DN favors a non-cytotoxic immune response in 5xFAD mice.

Other DAM-specific genes that were either not upregulated, or only slightly upregulated, in the cerebral cortex of 5xFAD/E2F4DN mice were *Axl* and *Csf1* [41] (Fig. 3c). AXL is a receptor tyrosine kinase crucial for the recognition of phosphoserine moieties in both apoptotic bodies and synapses to be pruned, and the subsequent activation of phagocytic capacity of microglia [45]. Therefore, the lack of *Axl* upregulation suggests a reduced phagocytic capacity of microglia in the cerebral cortex of 5xFAD/E2F4DN mice, which is consistent with the upregulation of *Anxa5* expression by E2F4DN (Fig. 3d), since this latter gene encodes Annexin V, a cytosolic protein that can suppress phagocytosis when extracellularly located, due to its capacity to interact with membrane phospholipids [46]. Furthermore, the lack of strong increase of *Csf1* in the cerebral cortex of 5xFAD/E2F4DN mice (Fig. 3c) is consistent with the attenuation of the inflammatory phenotype since colony stimulating factor 1, the *Csf1*-encoded protein, is a cytokine that stimulates phagocytic, cytotoxic, and chemotactic activity in macrophages [47].

qPCR also confirmed the upregulation of other relevant genes with the capacity to attenuate inflammatory response in the cerebral cortex of 5xFAD/E2F4DN mice. They include *Serpina3n* and *Spp1* (Fig. 3e). The former encodes a Granzyme B inhibitor that induces neuroprotection [48], while the latter encodes osteopontin (OPN), a tissue repair gene known to regulate immune cell function and to respond to brain injury [4]. OPN also modulates the ability of macrophage to resist pathogenic forms of Aβ [49]. Additionally, *Pglyrp1*, which encodes a peptidoglycan recognition protein that is expressed in polymorphonuclear leukocytes and is involved in antibacterial immunity and inflammation [50], is downregulated by E2F4DN (Fig. 3e).

### E2F4DN Expression Attenuates the Glial Response in 5xFAD Mice

**Fig. 4:**
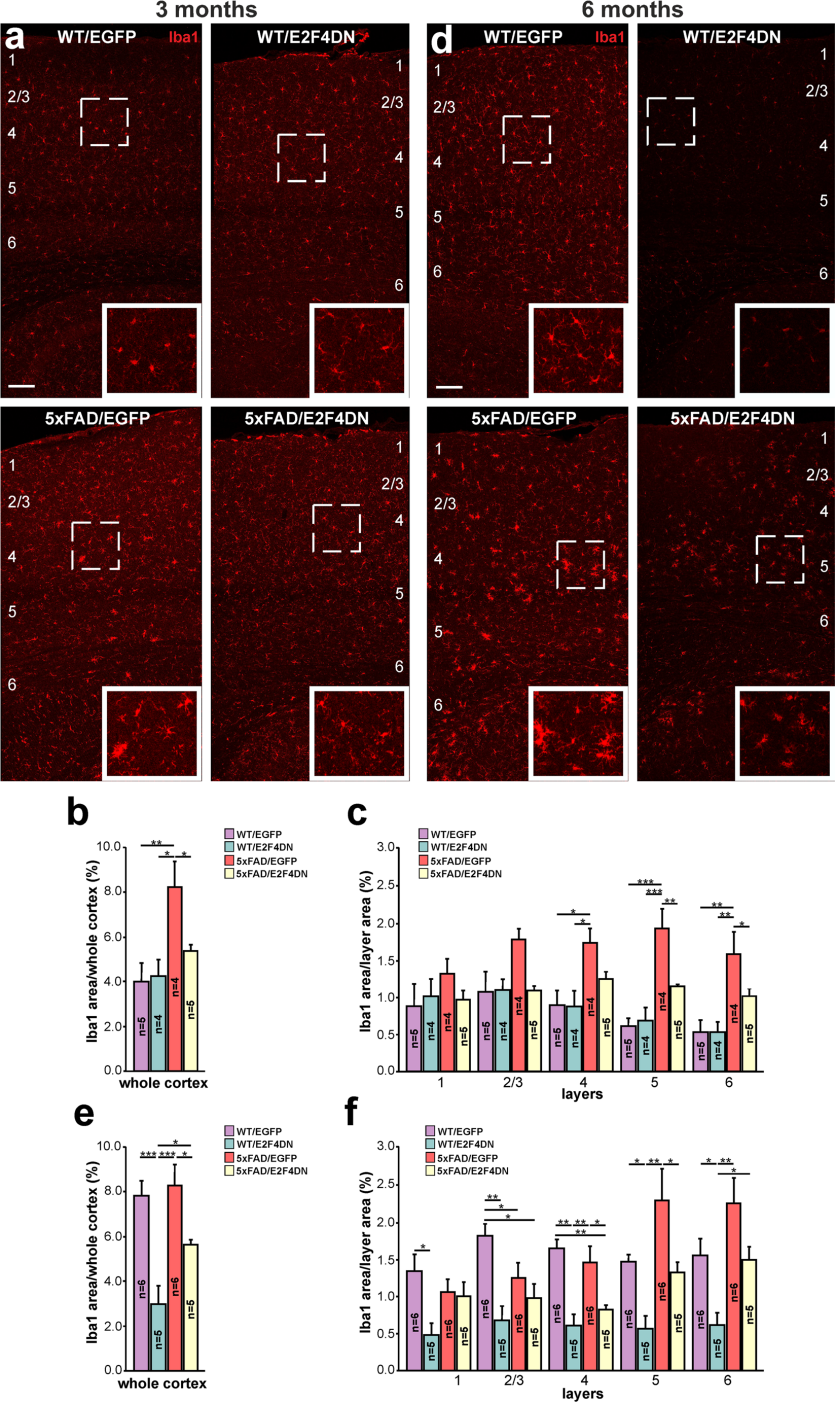
Modulation of microgliosis by E2F4DN in the cerebral cortex of 5xFAD mice. **a** Iba1 immunostaining in the cerebral cortex of mice of the indicated genotypes at 3 months (m) or 6 m. **b** Percentage of the area occupied by Iba1 immunostaining in the cerebral cortex of mice of the indicated genotypes at 3 m. **c** Percentages of the area occupied by Iba1 immunostaining in the indicated cortical layers at 3 m. **d** Iba1 immunostaining in the cerebral cortex of mice of the indicated genotypes at 6 m. Notice the decrease of Iba1-positive labeling in the cerebral cortex of 6 month-old WT mice expressing neuronal E2F4DN. **e** Percentage of the area occupied by Iba1 immunostaining in the cerebral cortex of mice of the indicated genotypes at 6 m. **f** Percentages of the area occupied by Iba1 immunostaining in the indicated cortical layers at 6 m. Numbers in **a** and **d** refer to the different cortical layers. Inserts in **a** and **d** show high magnifications of the indicated dashed boxes. *p < 0.05; **p < 0.01; ***p < 0.001 (one-way ANOVA followed by post hoc Newman-Keuls test). 3 months: WT/EGFP (1 male and 4 females), WT/E2F4DN (3 males and 1 female), 5xFAD/EGFP (3 males and 1 female), and 5xFAD/E2F4DN (4 males and 1 female). 6 months: WT/EGFP (4 males and 2 females), WT/E2F4DN (3 males and 2 females), 5xFAD/EGFP (3 males and 3 females), and 5xFAD/E2F4DN (5 males). Scale bar: 100 μm

To confirm that neuronal expression of E2F4DN favors an attenuated microglial response in the cerebral cortex of 5xFAD mice, we immunolabeled cortical sections of WT/EGFP, WT/E2F4DN, 5xFAD/EGFP, and 5xFAD/E2F4DN mice of 3 months with the specific microglia marker Iba1. This analysis demonstrated that, at this age, the area occupied by microglia in the cerebral cortex of 5xFAD/EGFP mice is significantly greater than that of WT/EGFP mice (Fig. 4a, b). This increase was mostly associated with layers 4–6 (Fig. 4c). This suggests that microglial cells are already activated in the cerebral cortex of 5xFAD/EGFP mice of 3 months of age even though no increase of immune markers is still observed in this tissue, when evaluated by qPCR (Fig. 3). In contrast, and consistent with the progressive activation of microglia with age [51], the area occupied by microglia in the cerebral cortex of WT/EGFP mice was greater at 6 months of age, reaching a similar area to that of microglial cells present in the cerebral cortex of 5xFAD/EGFP mice (Fig. 4d, e). This increase was equal in all cortical layers, compared to that is observed in the cerebral cortex of 5xFAD mice, where the increase in the area occupied by microglia was more prominent in layers 5–6 (Fig. 4f), possibly due to the control of the expression of APP and PS1 transgenes by the Thy1 promoter, which is mostly active in projection neurons from these layers [52].

The presence of E2F4DN significantly diminished the area occupied by microglial cells in 5xFAD mice at both 3 (Fig. 4a–c) and 6 months of age (Fig. 4d–f), supporting the hypothesis that neuronal E2F4DN attenuates the microgliosis observed in 5xFAD mice. Interestingly, E2F4DN was also able to prevent the increase in the area occupied by microglial cells in the cerebral cortex of WT/EGFP mice of 6 months of age (Fig. 4d–f).

A significant reduction of the area occupied by microglia was also evident in the hippocampus of 5xFAD/E2F4DN mice of 3 months, whereas just a tendency was noted at 6 months of age (Supplementary Fig. 4a, c).

**Fig. 5:**
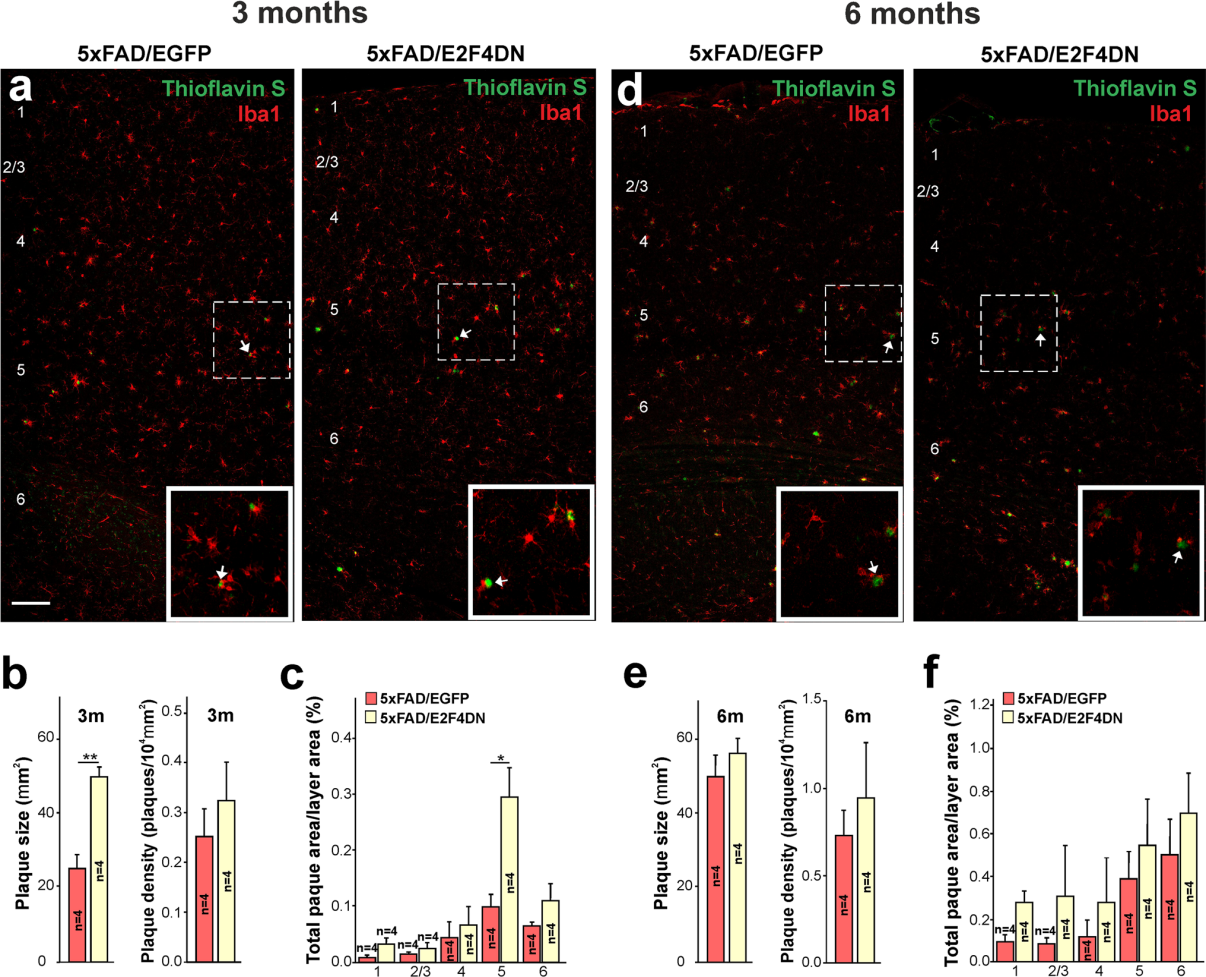
Modulation of Aβ deposition by E2F4DN in the cerebral cortex of 5xFAD mice. **a** Dense core plaques (Thioflavin S labeling, arrows) co-stained with Iba1 in the cerebral cortex of 3-month-old mice of the indicated genotypes. **b** Plaque size and plaque density in the cerebral cortex of 3-month-old mice of the indicated genotypes. **c** Percentages of the areas occupied by plaques in the indicated cortical layers at 3 months. **d** Thioflavin S labeling (arrows) co-stained with Iba1 in the cerebral cortex of 6 month-old mice of the indicated genotypes. **e** Plaque size and plaque density in the cerebral cortex of 6-month-old mice of the indicated genotypes. **f** Percentages of the areas occupied by plaques in the indicated cortical layers at 6 months. Numbers in **a** and **d** refer to the different cortical layers. Inserts show high magnifications of the indicated dashed boxes. *p < 0.05; **p < 0.01 (Student’s *t* test). 3 months: 5xFAD/EGFP (3 males and 1 female) and 5xFAD/E2F4DN (3 males and 1 female). 6 months: 5xFAD/EGFP (3 males and 1 females) and 5xFAD/E2F4DN (5 males). Scale bar: 100 μm

The hypothetical reduction of the phagocytic capacity of microglia, as suggested by the results obtained from our RNA-seq/qPCR analysis, was consistent with the increase in the size of the Aβ deposits observed at 3 months in the cerebral cortex of 5xFAD/E2F4DN mice, when compared to 5xFAD/EGFP control mice (Fig. 5a, b). Compared to their size, the density of the Aβ deposits was not significantly modified by the expression of E2F4DN (Fig. 5b) and, as expected, no plaques were detected in WT/EGFP and WT/E2F4DN mice (Supplementary Fig. 5). The latter correlated with the absence of Aβ production in WT control mice (Supplementary Fig. 6). The increase in size but not density of the Aβ deposits observed in the cerebral cortex of 5xFAD/E2F4DN mice, which was also observed in the hippocampus (Supplementary Fig. 4b, d), was most evident in the cortical layer 5 (Fig. 5c). The increase of plaque size was neither due to the intensification of Aβ production (Supplementary Fig. 7) nor the anomalous distribution of microglial cells, which were found surrounding the Aβ deposits in the 5xFAD/E2F4DN condition (Fig. 5a). Interestingly, the neuronal expression of E2F4DN slowed down Aβ accumulation in the cerebral cortex of 5xFAD mice at 6 months since their deposits did not increase in size at the same rate as in 5xFAD/EGFP control mice (Fig. 5d, e), and the area occupied in layer 5 by the Aβ deposits was similar in both genotypes (Fig. 5f). A similar effect was also observed in the hippocampus of 6 month-old 5xFAD/E2F4DN mice (Supplementary Fig. 4e). Therefore, neuronal E2F4DN expression was able to attenuate Aβ deposition at later stages of the AD pathology.

When activated, microglial cells increase their body size [36]. Morphological analysis of Iba1-positive cells from 5xFAD mice indicated that microglial cells in the cerebral cortex of 5xFAD/EGFP mice showed a more variable soma size when compared to 5xFAD/E2F4DN (Supplementary Fig. 8a, c), while neuronal expression of E2F4DN led to a non-significant tendency to reduce their body size at both 3 (Supplementary Fig. 7a) and 6 months of age (Supplementary Fig. 8c). These observations may be consistent with reduced microglia activation in the presence of neuronal expression of E2F4DN. No significant differences were observed when microglial roundness was evaluated (Supplementary Fig. 8b, d).

**Fig. 6:**
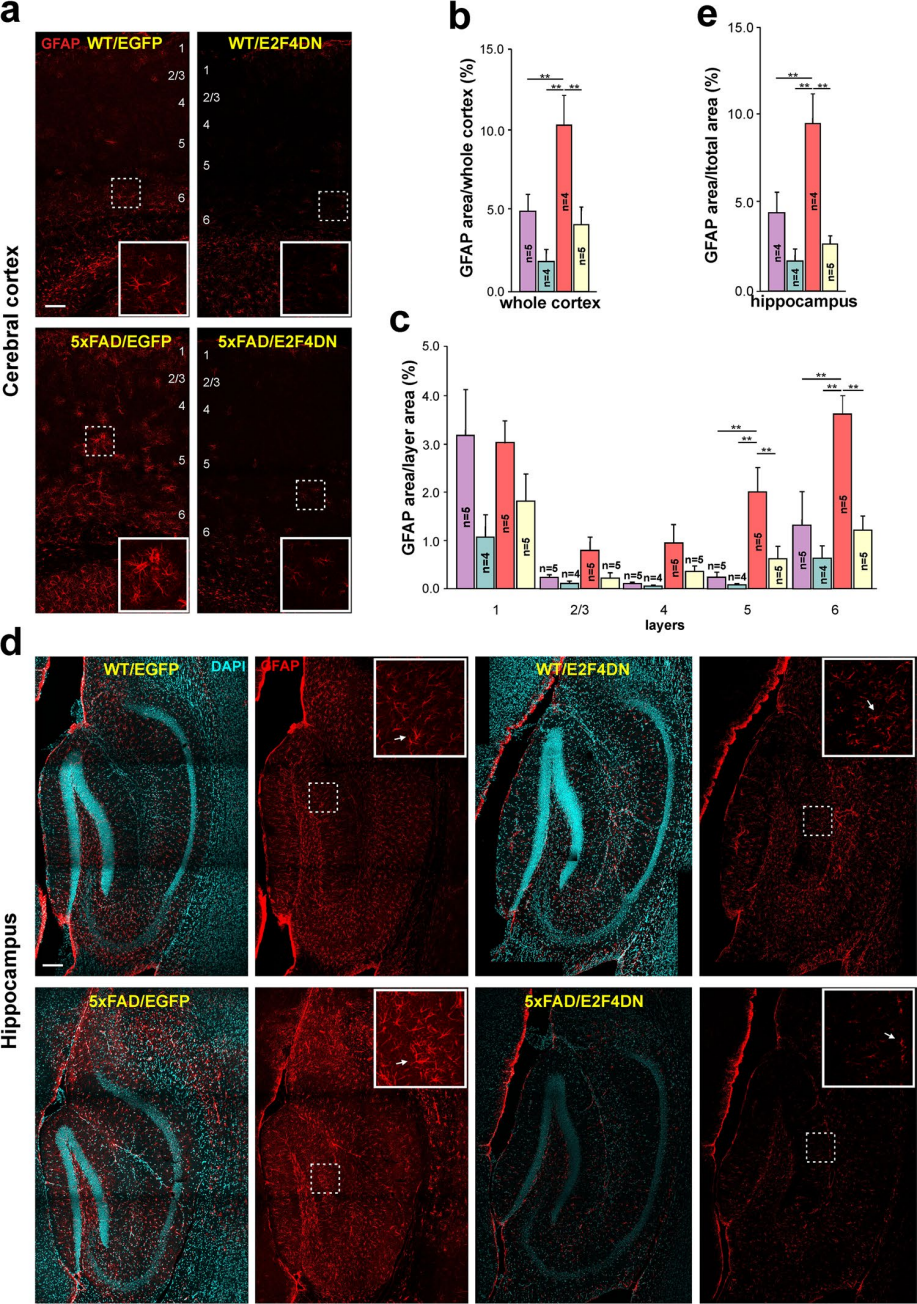
Modulation of astrogliosis by E2F4DN in cerebral cortex and hippocampus of 3 month-old 5xFAD mice. **a** GFAP immunostaining in the cerebral cortex of mice of the indicated genotypes. Notice the decreased GFAP-positive labeling and reactivity of astrocytes in the cerebral cortex of 5xFAD mice expressing neuronal E2F4DN. A similar trend, although not so pronounced, was also detected in WT mice expressing neuronal E2F4DN. Numbers refer to the different cortical layers. **b** Percentage of the area occupied by GFAP immunostaining in the cerebral cortex. **c** Percentages of the area occupied by GFAP immunostaining in the indicated cortical layers. **d** GFAP immunostaining in the hippocampus of mice of the indicated genotypes. Notice the decreased GFAP-positive labeling of astrocytes in the hippocampus of 5xFAD mice expressing neuronal E2F4DN. A similar trend, although not so pronounced, was also detected in WT mice expressing neuronal E2F4DN. **e** Percentage of the area occupied by GFAP immunostaining in the hippocampus. Inserts show high magnifications of the indicated dashed boxes. DAPI counterstaining is included to identify the hippocampus. **p < 0.01 (one-way ANOVA followed by post hoc Newman-Keuls test). Cortex: WT/EGFP (1 male and 4 females), WT/E2F4DN (3 males and 1 female), 5xFAD/EGFP (4 males and 1 female), and 5xFAD/E2F4DN (4 males and 1 female). Hippocampus: WT/EGFP (1 male and 3 females), WT/E2F4DN (3 males and 1 female), 5xFAD/EGFP (4 males) and 5xFAD/E2F4DN (4 males and 1 female). Scale bar: 100 μm

Neuroinflammation in 5xFAD mice is accompanied by reactive astrogliosis [53] starting at 3 months of age (see Supplementary Fig. 9). We therefore performed GFAP immunostaining in cerebral tissue from 5xFAD/EGFP and 5xFAD/E2F4DN mice of 3 months of age. This analysis indicated that the neuronal expression of E2F4DN reduces the area of GFAP immunoreactivity in the cerebral cortex of 5xFAD mice (Fig. 6a, b), mostly affecting layers 5 and 6. A significant reduction of GFAP immunostaining was also observed in the hippocampus (Fig. 6d, e). Interestingly, a non-significant trend to reduction of GFAP immunostaining could also be detected in the cerebral cortex and the hippocampus of WT mice (Fig. 6a–e). These results contrast with the increase of the *Gfap* transcript detected by RNA-seq (Supplementary Table 1) and qPCR in both cerebral cortex (Fig. 3f) and hippocampus (Supplementary Fig. 3c), suggesting that posttrancriptional mechanisms could regulate GFAP expression [54].

E2F4DN also induced a non-significant reduction of the area occupied by GFAP in the cerebral cortex of WT mice (Fig. 6b, c), as well as in the hippocampus (Fig. 6e), suggesting that neuronal expression of E2F4DN may also modulate age-associated astrocytosis [55].

We conclude that neuronal E2F4DN expression seems to favor a DAM-like phenotype with modulated phagocytic and cytotoxic capacity as well as reduced astrogliosis, which altogether may benefit neuronal welfare.

### E2F4DN Expression Controls Gene Networks Involved in Processing, Accumulation, and Toxicity of Aβ in 5xFAD Mice

**Fig. 7:**
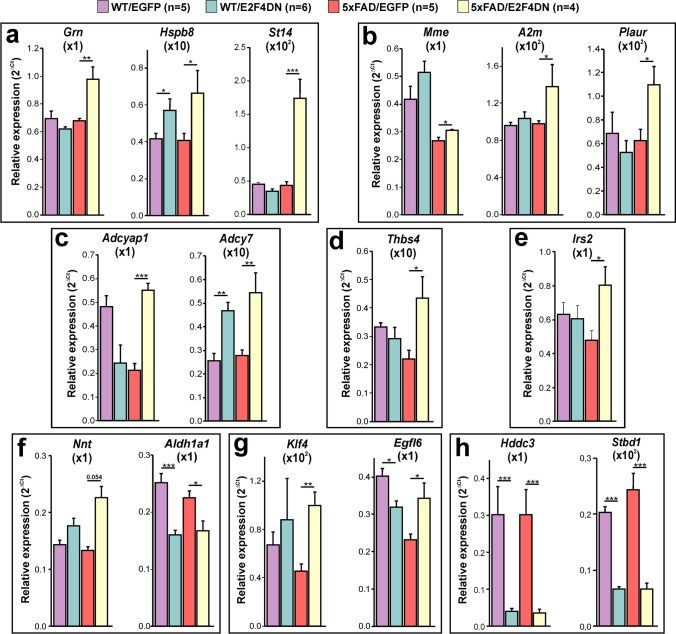
Gene expression in the cerebral cortex of 3 month-old mice of the indicated genotypes. qPCR analysis of genes preventing processing, accumulation and toxicity of Aβ (**a**); genes not detected by RNA-seq that are involved in Aβ aggregation and processing (**b**); genes involved in the PACAP signaling pathway (**c**), *Thbs4*, which regulates neurite outgrowth and synapse formation (**d**); *Irs2*, which regulates insulin signaling (**e**); genes involved in oxidative stress regulation (**f**); genes involved in vascular integrity (**g**); and brain welfare markers (**h**). Relative gene expression was normalized to *Rps18* rRNA levels and expressed as 2ΔCt (obtained values were adjusted by the factor indicated between brackets). WT/EGFP (5 females), WT/E2F4DN (3 males and 3 females), 5xFAD/EGFP (5 females), and 5xFAD/E2F4DN (4 females). *p < 0.05; **p < 0.01; ***p < 0.001 (Unbalanced two-way ANOVA, followed by post hoc Student’s *t* test)

Among the genes with increased expression in the cerebral cortex of 5xFAD/E2F4DN mice, a small group is involved in preventing the processing, accumulation and toxicity of Aβ (Fig. 7a). This group includes *Grn*, which encodes Progranulin, a neurotrophic growth factor that protects against Aβ deposition and toxicity [56]; *Hspb8*, which encodes a heat shock protein that inhibits Aβ aggregation and toxicity [57]; and *St14*, a microglial gene [40] that encodes Matriptase, a type II transmembrane serine protease that cleaves APP and reduces its processing to Aβ [58]. We selected *Hspb8* to confirm that the hippocampus shows a similar gene network (Supplementary Fig. 3d). The upregulation of other genes involved in Aβ aggregation and processing in the cerebral cortex of 5xFAD/E2F4DN mice was also demonstrated by qPCR (Fig. 7b). They include *Mme*, which encodes neprilysin, an Aβ-degrading enzyme [59]; *A2m*, which encodes α2-macroglobulin, an extracellular chaperone that inhibits amyloid formation [69]; and *Plaur*, which encodes a urokinase-type plasminogen activator receptor. This protein can be a protective factor for degradation and clearance of Aβ [61]. *Adcyap1*, which encodes pituitary adenylate cyclase activating polypeptide (PACAP), is also upregulated in the cerebral cortex of 5xFAD mice expressing neuronal E2F4DN (Fig. 7c). This neuropeptide, which is reduced in the brain of Alzheimer patients [62] and protects neurons against β-amyloid toxicity [62, 63], has been proposed as a therapy for AD [62]. PACAP has been shown to stimulate the non-amyloidogenic processing of APP and to increase the expression of BDNF and of the antiapoptotic Bcl-2 protein [63]. PACAP can also enhance the expression of the Aβ-degrading enzyme neprilysin in the mouse brain [63]. *Adcy7*, which encodes the major adenylate cyclase isoform downstream of PACAP [64], is also increased in the cerebral cortex of 5xFAD/E2F4DN mice (Fig. 7c). Overall, the observed gene network that enables reduced processing, accumulation, and toxicity of Aβ likely facilitates brain welfare while accounting for the size control of Aβ deposits in a genetic context supporting the reduced phagocytic capacity of microglia.

### E2F4DN Expression May Modulate Other Pathological Gene Networks in 5xFAD Mice

A number of differentially expressed genes, involved in functions that are relevant for AD, other than neuroinflammation or Aβ metabolism/toxicity, modulated their expression in the RNA-seq analysis (Supplementary Table 1). We selected a number of these genes as well as others with unclear effects on AD, to verify the predictive capacity of our RNA-seq analysis using real-time RT-PCR.

Among the first set of genes (i.e. those involved in AD relevant functions), we focused on *Irs2*, *Nnt*, *Aldh1a1*, *Klf4*, *Egfl6*, *Hddc3*, *Stbd1*, and *Thbs4*. *Irs2* encodes insulin receptor substrate 2, a mediator of the insulin signaling pathway, which is compromised in AD [65]. We confirmed that E2F4DN increases *Irs2* expression in 5xFAD mice (Fig. 7e). *Nnt* encodes nicotinamide nucleotide transhydrogenase, an integral protein of the inner mitochondrial membrane involved in antioxidant defense in this organelle [66], a crucial process in AD. This gene showed a tendency (*p* = 0.054) to become upregulated in the cerebral cortex of 5xFAD/E2F4DN mice (Fig. 7f). *Aldh1a1* encodes an aldehyde dehydrogenase that becomes upregulated under oxidative stress conditions [67]. This gene was observed to become downregulated in the cerebral cortex of 5xFAD/E2F4DN mice (Fig. 7f). *Klf4*, which encodes a protein that potentiates endothelial and vascular integrity [68], was also confirmed to become upregulated (Fig. 7g). *Egfl6*, which encodes an extracellular matrix protein involved in angiogenesis [69], was confirmed to decrease its expression in 5xFAD control mice while becoming upregulated by E2F4DN. Metabolic stress response markers including *Hddc3*, a putative marker of cell starvation [70], and *Stbd1*, a glycophagy marker [71], were also confirmed to be downregulated (Fig. 7h). Additionally, we focused on *Thbs4*, which encodes Thrombospondin 4, a member of the thrombospondin family that regulates neurite outgrowth and synapse formation, and has been related to AD [72]. We confirmed that this gene becomes upregulated by E2F4DN in 5xFAD mice (Fig. 7d). The upregulation of *Thbs4* gene expression suggests that synaptic function is potentiated in 5xFAD mice expressing neuronal E2F4DN. Accordingly, we found that synaptophysin, a known marker used for synapse quantification [73], is upregulated in the hippocampus of 5xFAD/E2F4DN mice (Supplementary Fig. 10). Finally, we found that a selected subset of the genes described above show a similar modulation by E2F4DN in the hippocampus (Supplementary Fig. 3e-f).

Among other genes whose expression is modulated by E2F4DN with unclear purpose are *Adi1*, *Barx2*, *Cfap46*, and *Cfap54*. *Adi1*, which is upregulated in the cerebral cortex of 5xFAD/E2F4DN mice (Supplementary Fig. 11a), encodes acireductone dioxygenase 1, an enzyme that participates in the metabolism of methionine and is downregulated in subjects with AD [74]. *Barx2*, which is a member of the homeobox transcription factor family, was also confirmed as downregulated in the cerebral cortex of 5xFAD/E2F4DN mice (Supplementary Fig. 11a). Finally, *Cfap46* and *Cfap54*, which encode two structural proteins from motile cilia [75], were confirmed as having opposed modulation by E2F4DN (Supplementary Fig. 11b).

### E2F4DN Expression Prevents Neuronal Tetraploidization in the Cerebral Cortex of 5xFAD Mice

**Fig. 8:**
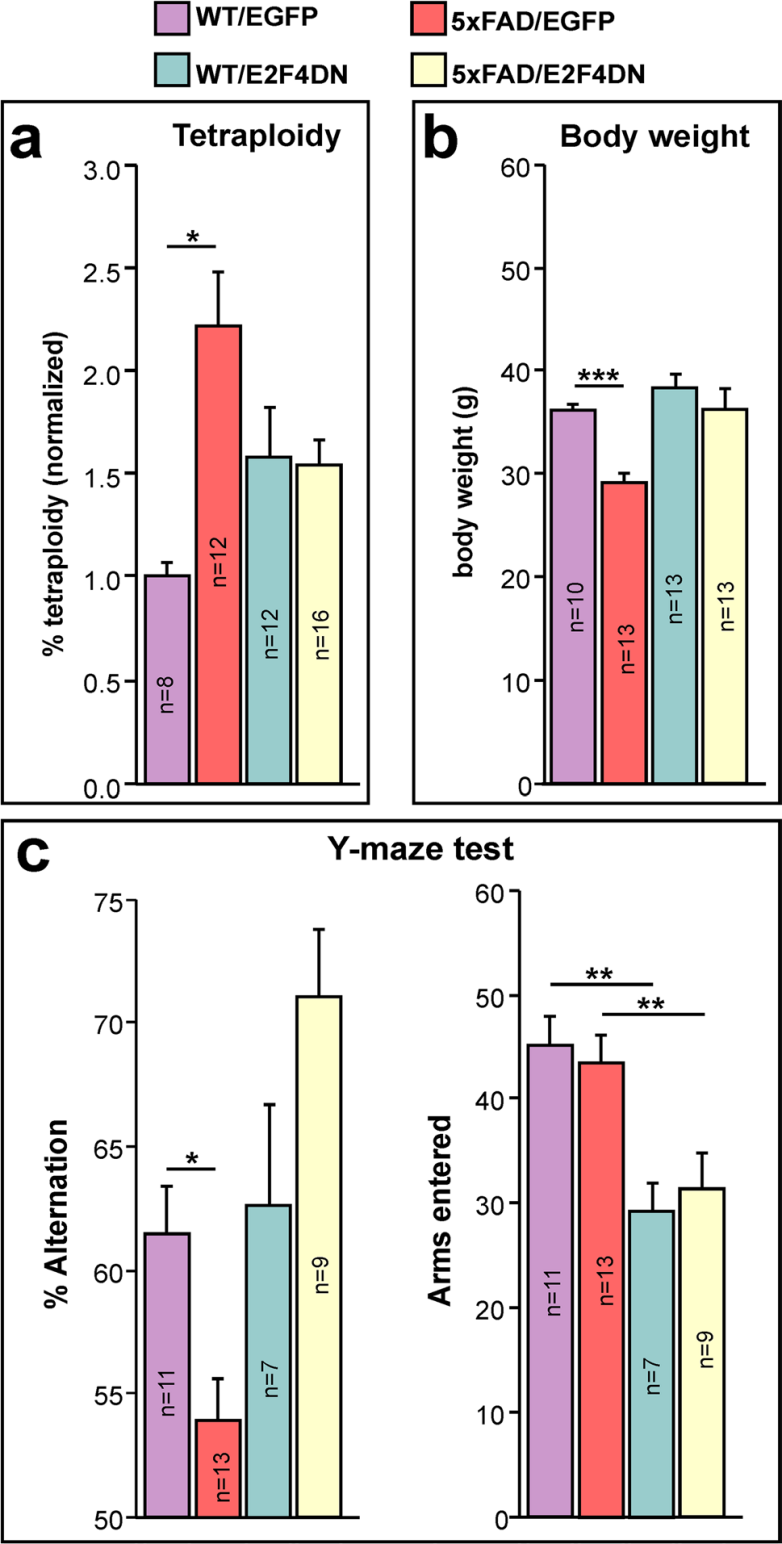
Effects of E2F4DN on NT, body weight, and spatial working memory in 5xFAD mice. **a** NT quantification, normalized to NT levels in the cerebral cortex of 2-month-old WT mice, in cell nuclear extracts from cerebral cortex of 3-month-old littermates from crosses between 5xFAD transgenic mice and either homozygous EGFP mice or E2F4DN mice. No significant differences between WT/EGFP and WT/E2F4DN were observed (*p* = 0.114). WT/EGFP (5 males and 3 females), WT/E2F4DN (4 males and 8 females), 5xFAD/EGFP (6 males and 6 females), and 5xFAD/E2F4DN (1 male and 15 females). **b** Body weight in one-year-old male littermates from crosses between 5xFAD transgenic mice and either homozygous EGFP mice or homozygous E2F4DN mice. **c** Left: Percentage of alternation observed in 5-month-old male littermates from crosses between 5xFAD transgenic mice and either homozygous EGFP mice or homozygous E2F4DN mice. Random alternation (50%) indicates full memory loss. E2F4DN expression prevents spatial working memory impairment in 5xFAD mice as evaluated by the spontaneous alternation Y-maze test. Right: Number of arms entered by 5-month-old littermates from crosses between 5xFAD transgenic mice and either homozygous EGFP mice or homozygous E2F4DN mice. *p < 0.05; ***p < 0.001 (unbalanced two-way ANOVA, followed by post hoc Student’s *t* test)

Neuronal tetraploidy could be an important etiological factor in AD [38]. To study whether neuronal expression of E2F4DN can prevent AD-associated NT, we analyzed this parameter in descendants of crosses between 5xFAD mice and either E2F4DN or EGFP mice at 3 months of age. The proportion of tetraploid nuclei for each genotype was normalized to the value obtained in the cerebral cortex of WT mice of 2 months of age [37], which was used as an internal reference in all the experiments [38]. These analyses indicated that NT was increased in 5xFAD/EGFP mice when compared with WT/EGFP littermates (Fig. 8a), in agreement with previous observations in APP/PS1 mice [38]. This effect was blocked in the presence of E2F4DN (Fig. 8a). We therefore conclude that E2F4DN is able to prevent NT, as expected from its capacity to prevent cell cycle re-entry in differentiating chick retinal neurons [23].

### E2F4DN Expression Prevents Body Weight Loss in 5xFAD Mice

A previous report demonstrated that 5xFAD mice show progressive body weight loss, starting at 9 months of age [76], a pathological effect also observed in AD patients [1]. In accordance with Jawhar et al. [74], we found a loss of body weight when 5xFAD/EGFP mice were compared to WT/EGFP mice at 1 year of age (Fig. 8b). Conversely, no body weight loss was detected in the presence of E2F4DN (Fig. 8b). This indicates that the expression of E2F4DN can reverse this somatic phenotype in 5xFAD mice.

### E2F4DN Expression Prevents Spatial Memory Deficits in 5xFAD Mice

5xFAD mice of 4–5 months of age display cognitive impairment, as evidenced by the spontaneous alternation Y-maze paradigm [26] and by cognitive tasks evaluating reference memory (MWM test). We therefore tested whether E2F4DN expression reverses this phenotype. To this end, spontaneous alternation performance in the Y-maze test was analyzed in 5-month-old descendants from both 5xFAD/EGFP and 5xFAD/E2F4DN crosses. This analysis confirmed that 5xFAD mice show reduced probability of alternation under control conditions (Fig. 8c, left). Comparatively, the expression of E2F4DN in neurons fully reversed the spatial memory deficits of 5xFAD mice (Fig. 8c, left). These results were independent of the number of arms that were entered in either WT/EGFP vs. 5xFAD/EGFP or WT/E2F4DN vs. 5xFAD/E2F4DN mice (Fig. 8c, right), although a statistically significant reduction of this parameter was detected in mice carrying the E2F4DN allele. Therefore, working memory is improved in 5xFAD mice with neuronal expression of E2F4DN.

The presence of neuronal mE2F4DN-myc modestly prevented spatial, hippocampal-depending learning impairment, previously described in 5xFAD mice during the training period in the MWM paradigm [77]. Six-month-old descendants from both 5xFAD/EGFP (Supplementary Fig. 13a) and 5xFAD/mE2F4DN-myc (Supplementary Fig. 13b) crosses were challenged to swim until they found a platform hidden below the water level. This task was repeated four times daily for five consecutive days. As expected, WT mice expressing neuronal EGFP showed a rapid decrease in the escape latency that could not be mimicked by their 5xFAD littermates until the third day of training (Supplementary Fig. 13a). In contrast, the presence of neuronal mE2F4DN-myc resulted in non-significant differences in escape latencies between WT and 5xFAD mice at any experimental time point (Supplementary Fig. 13b). Altogether, these results indicate that both spatial learning and working memory are improved in 5xFAD with neuronal expression of mE2F4DN-myc. These effects do not rely on better locomotor activity, as evidenced by the activity cage test (Supplementary Fig. 12a, b), or motor coordination, as measured by a rotarod test (Supplementary Fig. 12c).

### E2F4 is Expressed in Cortical Neurons of AD Patients

To provide support to E2F4DN as a potential therapeutic agent, we studied whether E2F4 is present in human cortical neurons from Alzheimer patients and whether it is associated to Thr phosphorylation. To this end, we performed PLA [78] in cryosections from parietal cortex of Alzheimer patients at Braak stages I and VI [79] with two different antibodies against E2F4. This analysis demonstrated that E2F4 is expressed in neurons at both stages, as revealed by NeuN-specific immunostaining (Supplementary Fig. 14a, b). Additionally, using the PLA method with an anti-E2F4 together with an anti-phosphoThr antibody demonstrated that the labeling of E2F4 in cortical neurons is associated with Thr-specific phosphorylation in AD patients at Braak stage I, and this association is maintained at Braak stage VI (Fig. 14a, c). Therefore, our PLA results demonstrate that E2F4 is present in AD-associated cortical neurons and they suggest that, in these cells, E2F4 is phosphorylated in Thr even at the earliest stages of the disease, before the presence of NFTs are visible in the parietal cortex [79].

## Discussion

Although E2F4 has been recognized for decades as a transcription factor with a crucial role in the control of cell quiescence, recent evidence indicates that it can also fulfill multiple homeostatic functions [80]. In the Aβ-stressed neurons, E2F4 could play a protective role [81] due to its potential capacity to bind the regulatory domains of over 7,000 genes and to modulate several transcriptional networks involved in cell stress response [21].

Our results support this hypothesis since the mutation of the conserved Thr249/T251 motif of mouse E2F4, which is susceptible to phosphorylation [22], is crucial for maintaining neuronal quiescence and brain homeostasis even in the presence of elevated levels of Aβ. This is consistent with our previous observation that E2F4 is associated with phosphoThr immunoreactivity in cortical neurons from APP/PS1 mice [25], a finding that can be expanded to parietal neurons from AD patients as shown in this study. These results, based on PLA, a method previously used to study the phosphorylation status of proteins [35], suggest that the phosphorylation of E2F4 in Thr residues can participate in the etiology of AD.

In stressed neurons, E2F4 phosphorylation could induce cell cycle progression [23, 82] and a hypothetical deregulation of cell cycle-independent gene expression involved in the etiology of AD, thus explaining the capacity of E2F4DN to restore normal E2F4 function and to prevent several AD-associated processes. Although the conserved Thr249/Thr251 motif can be phosphorylated by p38^MAPK^ [23], a stress kinase upregulated in AD [24], it cannot be ruled out that other related stress kinases may also lead to E2F4 phosphorylation in AD.

We have found that neuronal E2F4DN expression results in reduced astrogliosis and microgliosis in both WT and 5xFAD mice. Microglial cells function as a sensor of changes in their environment and respond to such changes, thus providing neuroprotection, while an exacerbation of this essential function leads to neurodegeneration. Correcting this imbalance may be a potential mode for therapy [83]. We have shown that the neuronal expression of E2F4DN modulates both the area occupied by Iba1-specific labeling and the expression of genes involved in microglial function, likely through well-established mechanisms of bidirectional neuron-glia communication. This includes neuronal immunomodulators, such as CX3CL1 and CD200, the neuron-derived factor CD22, the signal regulatory protein α (SIRPα), CD47, and neurotransmitters such as norepinephrine and ATP and its metabolites adenosine diphosphate and adenosine [84]. Nevertheless, the participation of neuronal exosomes [85] cannot be ruled out, as suggested by the gene ontology analysis shown in Supplementary Table 2. The resulting microglial phenotype might favor brain homeostasis reducing adverse effects derived from cytotoxicity and Aβ phagocytosis [4, 86]. We have also shown that neuronal expression of E2F4DN modulates astrocytosis. This effect could be mediated by well-known neuron-to-astrocyte signaling dependent on neuronal activity [87] or endocannabinoids [88].

ApoE is mainly upregulated by microglia in response to Aβ deposition, and plays a critical role in the ability of microglia to take up and degrade Aβ [89]. Therefore, the observation that *Apoe* expression remains at low levels in the cerebral cortex of 5xFAD/E2F4DN mice, while other DAM-associated genes become strongly upregulated is consistent with a putative DAM phenotype with reduced Aβ phagocytic capacity and diminished expression of inflammatory cytokines (5). This hypothesis would be consistent with the decreased Iba1-positive area observed in h5xFAD mice expressing neuronal E2F4DN. A reduced clearance of fibrillar Aβ by microglia could explain the accumulation of Aβ dense core deposits, as evidenced by Thioflavin S labeling, which is observed at 3 months in 5xFAD/E2F4DN mice, and whose deleterious effects are likely attenuated by the expression of a repertoire of genes that prevent Aβ processing, accumulation and toxicity. Interestingly, Aβ deposition was attenuated by E2F4DN in 6 month-old 5xFAD/E2F4DN mice. This observation may be explained by the lack of strong *Apoe* overexpression since ApoE potentiates fibrillation and compactation of Aβ at later pathological stages, when amyloid plaque growth becomes independent of TREM-2 [89].

Other pathological mechanisms could also be attenuated by E2F4DN in 5xFAD mice, as relevant genes involved in the complex etiology of AD were found to be positively modulated. This includes regulators of synaptic function, glucose metabolism, oxidative stress, and endothelial cell function. The demonstration that NT is prevented by neuronal E2F4DN expression is also relevant. Cell cycle reentry has been proposed as a major driver of AD [10], likely due to its capacity to induce NFTs, extracellular deposits of Aβ, gliosis, synaptic dysfunction, and delayed neuronal cell death [12]. Furthermore, NT triggers synaptic dysfunction [90] and may affect neuronal structure and function [12]. Our results are consistent with the observation that prevention of NT in the cerebral cortex of aged E2f1^−/−^ mice correlates with enhanced cognition [37].

E2F4DN expression in neurons was able to reverse the body weight loss phenotype observed in 5xFAD mice [76]. Weight loss is a common symptom of AD [1], likely associated to metabolic alterations [91]. This latter view is consistent with the increase of the resting metabolic rate observed in a mouse model of tau deposition [92], as well as the early metabolic deficits detected in transgenic mice overexpressing APP, in association with hypothalamic dysfunction [93]. It is currently uncertain whether the effect of E2F4DN on AD-associated metabolic alterations is directly due to a hypothetical capacity to block NT in neurons involved in sensing leptin, an adipocytokine that regulates energy metabolism and appetite [94]. Alternatively, E2F4DN might act as a transcription factor on metabolism-regulating pathways. Indeed, E2F4 is known to be regulated by insulin signaling in preadipocytes [95]. Therefore, E2F4 seems to be linked to multiple pathways involved in obesity and energy metabolism, and this property may underscore the capacity of E2F4DN to reverse weight loss in 5xFAD mice without leading to obesity in WT mice.

In this study, we have demonstrated that E2F4DN expression prevents spatial learning deficits observed in 5xFAD mice, evaluated with the spontaneous alternation Y-maze test [26]. This effect was observed even though Aβ deposition was not fully abolished by E2F4DN, supporting previous studies demonstrating that cognition is compatible with the presence of extensive Aβ deposition in individuals with asymptomatic AD [27]. Therefore, Aβ seems to be necessary but not sufficient for the etiology of AD, where microglia plays a prominent role [4]. As in our study, others have shown strong Aβ accumulation in a clinical case with resistance to familial AD in correlation with an APOE3 mutation in homozygosis [96], which led the authors to propose that reduced ApoE activity is likely to prevent cognitive deficits.

Since Aβ deposition was not fully prevented by neuronal expression of E2F4DN, cognitive recovery in 5xFAD mice cannot simply result from Aβ accumulation blockage. The latter has been claimed as the reason why therapeutic approaches aimed at reducing amyloid burden that work in transgenic mice are not effective when translated to AD patients [97]. Conversely, our results suggest that E2F4DN is useful as a multifactorial therapeutic agent for AD. When E2F4DN is expressed in neurons, multiple neuropathological and somatic alterations observed in 5xFAD mice become attenuated without triggering major side effects. The absence of side effects was expected, as E2F4 is already expressed in neurons from 5xFAD mice. A major challenge for E2F4DN as a therapeutic agent is the method for its in vivo delivery in both AD mouse models and AD patients. We propose E2F4DN-based gene therapy as a realistic approach. Gene therapy is usually regarded as a process whereby WT genes are delivered in a tissue to replace abnormal genes that cause pathological effects. In our case, functional recovery would be obtained by expressing an E2F4 variant unable to become Thr phosphorylated, thus counteracting the pathological environment that favors E2F4 phosphorylation. Since E2F4 is already expressed by AD-affected neurons, it is conceivable that neuronal expression at physiological levels of E2F4DN should not trigger side effects in AD patients. Therefore, neuronal E2F4DN expression could efficiently target the complex etiology of AD, and thus become a promising molecule for successful therapy against this devastating disease.

AD is a cruel and devastating disease for which no effective therapies are currently available, possibly due to its multifactorial etiology. The findings of this study provide proof of concept that E2F4DN could be used as a therapeutic agent for the remediation of molecular, cellular and behavioral alterations underlining the pathophysiology of this disease. We propose E2F4DN-based gene therapy as a promising multifactorial approach against AD.

## Supplementary Information

ESM 1 (DOCX 3.41 MB)

ESM 2 (pptx 1.55 MB)

ESM 3 (xlsx 58.0 KB)

ESM 4 (xlsx 145 KB)

ESM 5 (xlsx 11.4 KB)

ESM 6 (xlsx 83.6 KB)

## Data Availability

The RNA sequencing dataset supporting the conclusions of this article is available in the SRA repository (http://www.ncbi.nlm.nih.gov/bioproject/725837). Other data generated or analyzed during this study are included in this published article and its supplementary information files.
