# Supplementary material for: A Mutant Variant of E2F4 Triggers Multifactorial Therapeutic Effects in 5xFAD Mice
Source: Mol Neurobiol. 2022 Mar 7;59(5):3016–39. doi: 10.1007/s12035-022-02764-z (PMC9016056; doi:10.1007/s12035-022-02764-z)
Supplement: Supplementary file 1 — (DOCX 3.41 MB) [file 12035_2022_2764_MOESM1_ESM.docx]

SUPPLEMENTARY INFORMATION

A mutant variant of E2F4 triggers multifactorial therapeutic effects in 5xFAD mice

Noelia López-Sánchez^1,^* · Morgan Ramón-Landreau^1,^* · Cristina Trujillo^1^ · Alberto Garrido-García^1^ · José M. Frade^1^

^1^Department of Molecular, Cellular and Developmental Neurobiology, Cajal Institute, Consejo Superior de Investigaciones Científicas, E-28002 Madrid, Spain

*Both authors contributed equally to this study

Correspondence: José M. Frade

frade@cajal.csic.es

**Supplementary Table 1** Differentially expressed genes in the cerebral cortex of 5xFAD/E2F4DN and 5xFAD/EGFP mice. This includes the *Egfp* and *E2f4*-derived transgenes, 21 non-coding RNAs, 2 microRNAs, 2 transcripts encoding uncharacterized proteins, and 2 sets of related gene sequences encoding immunoglobulin heavy constant and T cell receptor α constant and joining moieties. A non-exhaustive list of references that associated the indicated genes with AD is included.

**Supplementary Table 2** Bioinformatics analyses performed with differentially expressed genes in the cerebral cortex of 5xFAD/E2F4DN and 5xFAD/EGFP mice. **Differentially-expressed genes:** genes shown in Supplementary Table 1 (*Trac* and *Igha* were included as representative for the sequences encoding immunoglobulin heavy constant and T cell receptor α constant and joining moieties). Functional GO term annotation for **Biological Processes** (GOTERM_BP_ALL), **Cellular Components** (GOTERM_CC_ALL) and **Molecular Functions** (GOTERM_MF_ALL) are shown.

**Supplementary Table 3** Upregulated genes in the cerebral cortex of APP/PS2 transgenic mice, expressed in microglia, astrocytes, and neurons, as described by [41]. Pink: Genes common between APP/PS2 and 5xFAD/E2F4DN. Blue: Genes in APP/PS2 but not in 5xFAD/E2F4DN.

**Supplementary Table 4** Bioinformatics analyses performed with differentially expressed genes in the cerebral cortex of 5xFAD/E2F4DN and 5xFAD/EGFP mice as compared with upregulated genes in the cerebral cortex of APP/PS2 transgenic mice. **Common Genes** Functional GO term annotation for biological processes (GOTERM_BP_ALL), from microglia-expressed genes common to 5xFAD/E2F4DN and APP/PS2 mice [41]. **APP_PS2 Unique Genes** Functional GO term annotation for biological processes (GOTERM_BP_ALL) from microglia-specific genes unique in APP/PS2 mice [41]. **MGI Mammalian Phenotype** MGI Mammalian Phenotype database browsed against the protein-encoding genes modulated by E2F4DN in the cerebral cortex of 5xFAD mice that are absent in the study by [41]. *Trac* and *Igha* were included as representative for the sequences encoding immunoglobulin heavy constant and T cell receptor α constant and joining moieties shown in Supplementary Table ~~S~~1.


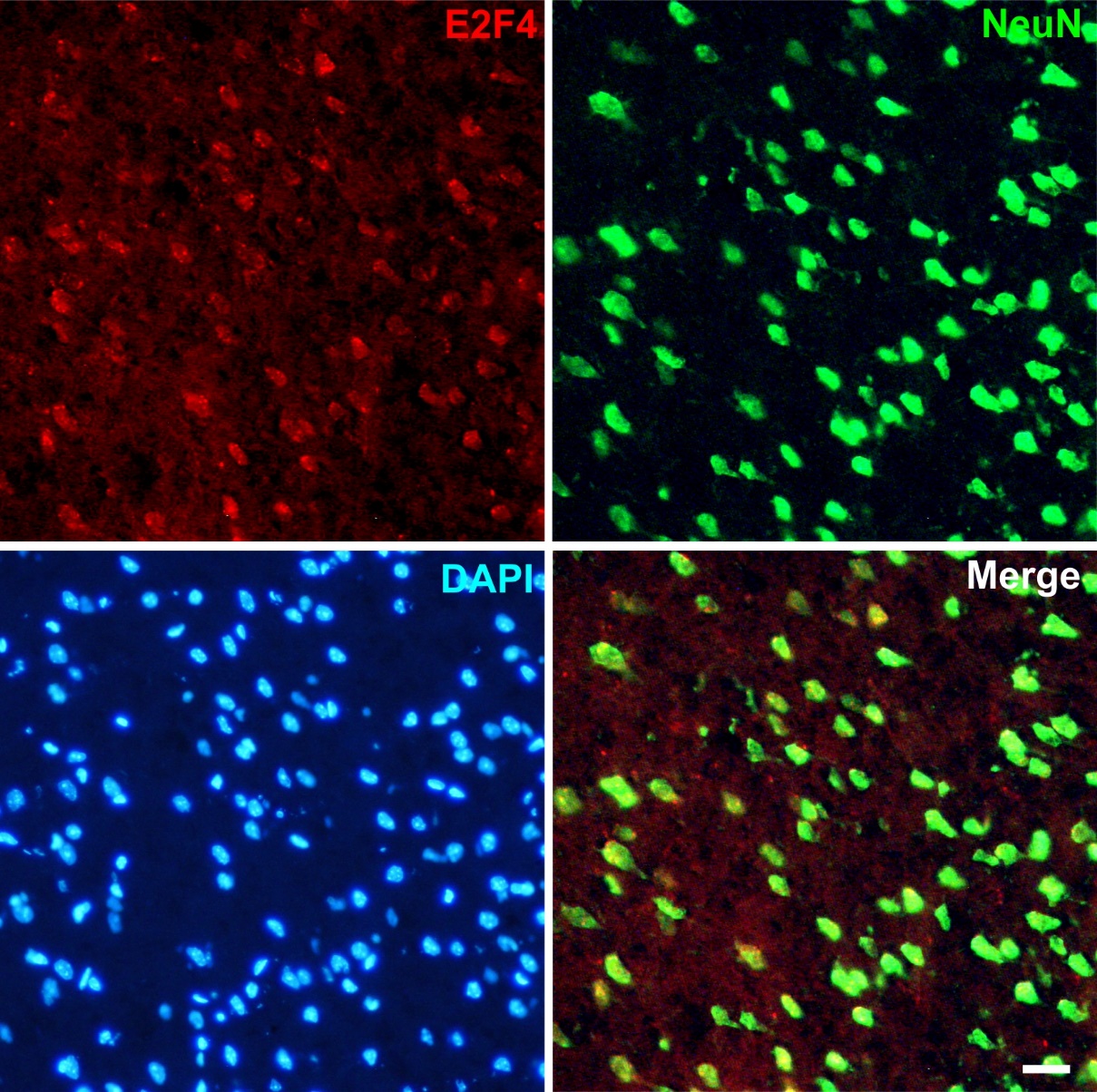


**Supplementary Fig. 1** E2F4 expression (red) in cortical neurons (NeuN+ cells) (green). Nuclei are labeled with DAPI (blue). Bar: 80 μm.

**
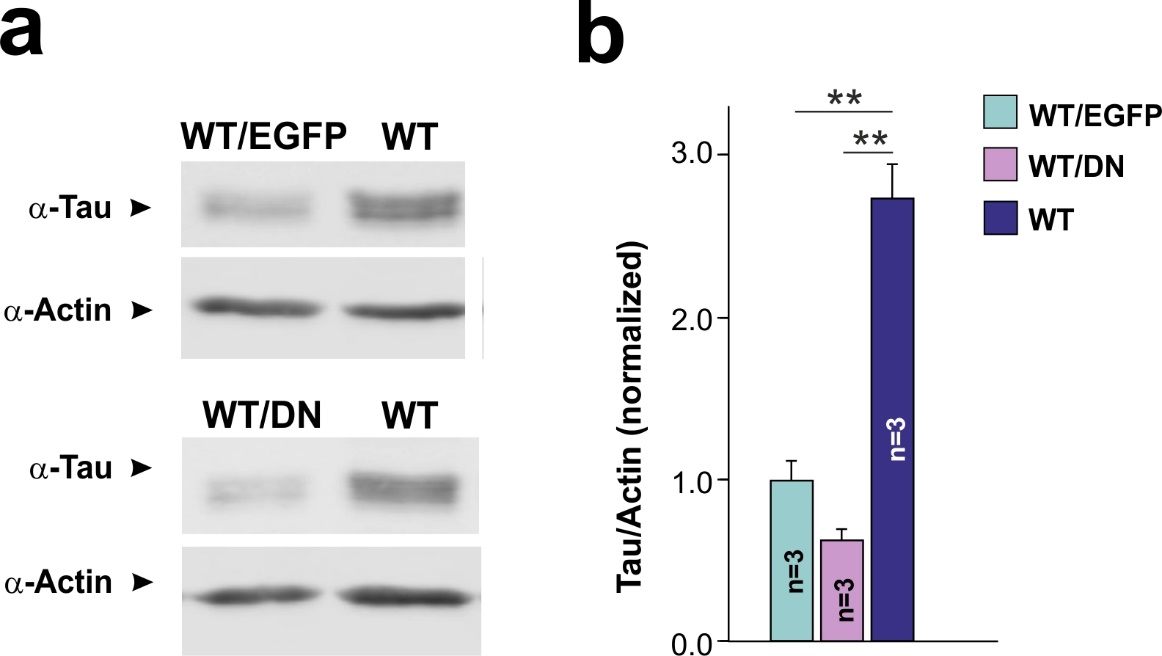
**

**Supplementary Fig. 2** Mice carrying a transgene within the *Mapt* locus show reduced expression of tau protein. **a.** Western blot analysis of cortical extracts from 3 month-old mice from the indicated genotypes (n=3 females/genotype) using antibodies against tau protein and actin (as a loading control). b. Quantification of the ratios of tau vs actin in the hippocampus from the indicated genotypes. DN: E2F4DN. **p<0.01 (Student’s *t* test).


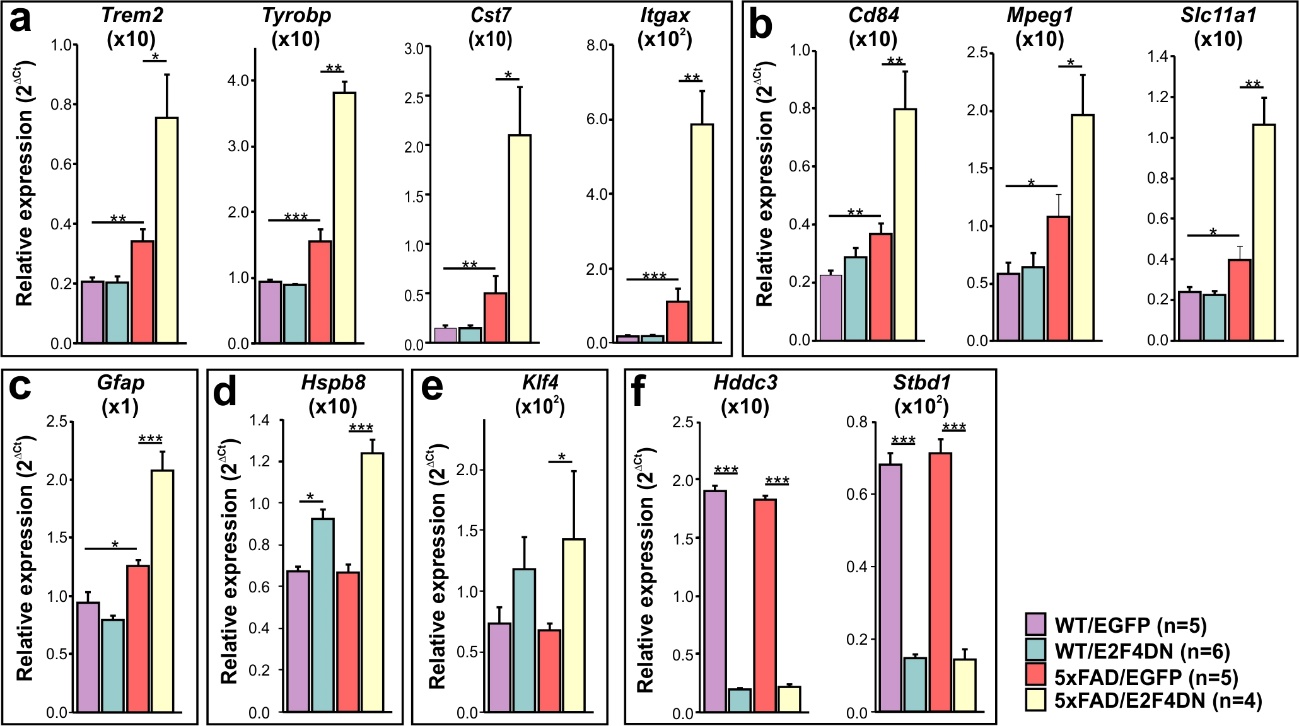


**Supplementary Fig. 3** Gene expression analysis by qPCR of select genes in the hippocampus of 3 month-old mice of the indicated genotypes (WT/EGFP: 5 females, WT/E2F4DN: 3 males and 3 females, 5xFAD/EGFP: 5 females, and 5xFAD/E2F4DN: 4 females). They include DAM-specific genes (**a**), innate immune response genes (**b**), *Gfap*, an astrocyte marker (**c**), *Hspb8*, which encodes a heat shock protein that inhibits Aβ aggregation and toxicity (**d**), *Klf4*, a gene involved in vascular integrity (**e**), and brain welfare markers (**f**). Relative gene expression was normalized to *Rps18* rRNA levels and expressed as 2ΔCt (obtained values were adjusted by the factor indicated between brackets). *p<0.05; **p<0.01; ***p<0.001 (unbalanced two-way ANOVA, followed by Student’s *t* test).


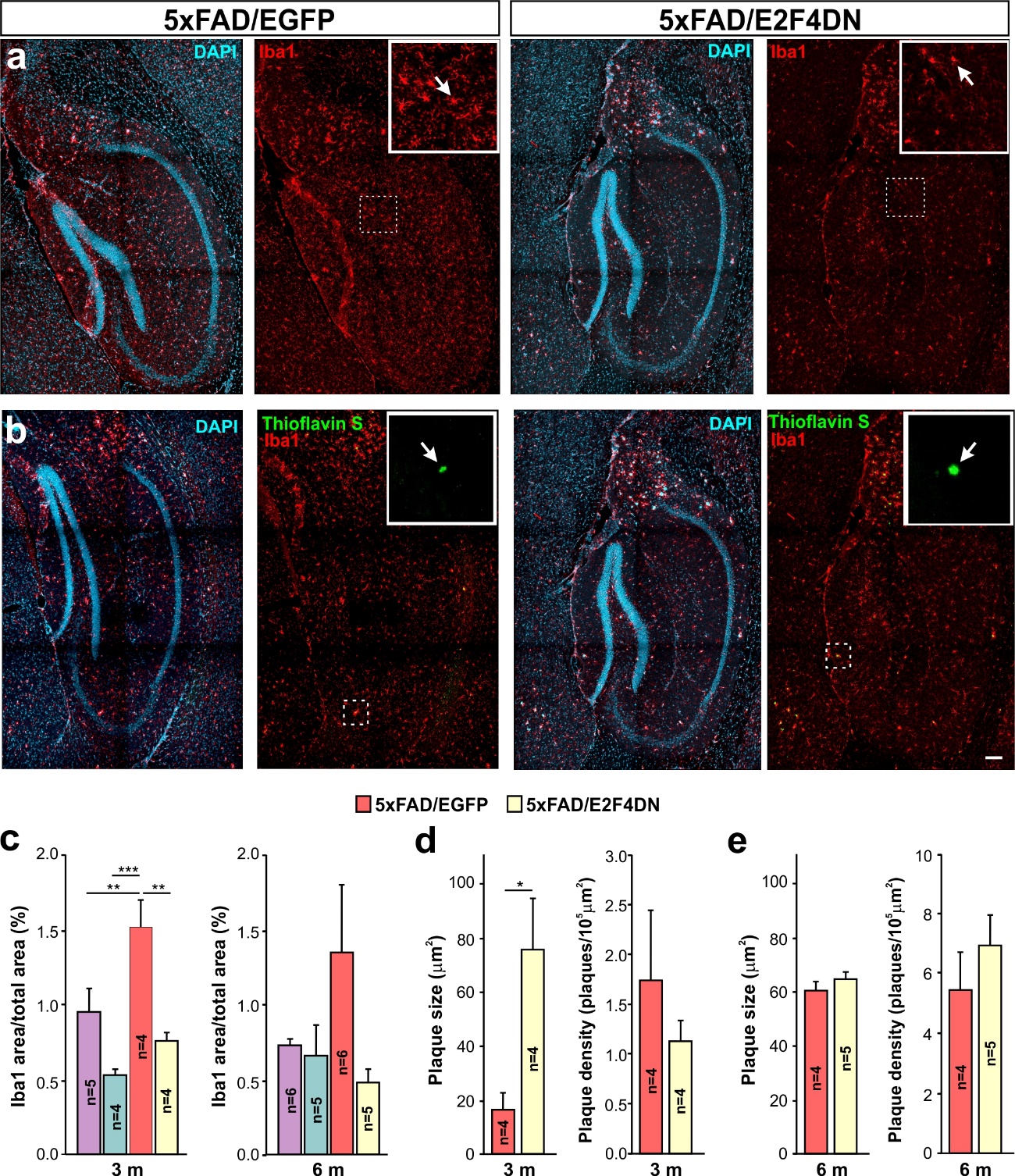


**Supplementary Fig. 4** Attenuation of microgliosis and modulation of Aβ deposition by E2F4DN in the hippocampus of 5xFAD mice. **a** Iba1 immunostaining in the hippocampus of mice of the indicated genotypes at 3 months of age. **b** Thioflavin S/Iba1 co-labeling in the hippocampus of mice of the indicated genotypes at 3 months of age. **c** Percentage of the area occupied by Iba1 immunostaining in the hippocampus at 3 months (3 m) and 6 months (6 m) of age. 3 months: 5xFAD/EGFP (3 males and 1 female) and 5xFAD/E2F4DN (3 males and 1 female). 6 months: 5xFAD/EGFP (3 males and 3 females) and 5xFAD/E2F4DN (5 males). **d** Plaque size and plaque density in the hippocampus of the indicated genotypes at 3 m: 5xFAD/EGFP (3 males and 1 female) and 5xFAD/E2F4DN (3 males and 1 female). **e** Plaque size and plaque density in the hippocampus of the indicated genotypes at 6 m: 5xFAD/EGFP (3 males and 1 females) and 5xFAD/E2F4DN (5 males). Inserts show high magnifications of the indicated dashed boxes. DAPI counterstaining is included to identify the hippocampus. *p<0.05; **p<0.01 (one-way ANOVA, followed by *post hoc* Newman-Keuls for the percentage of the area occupied by Iba1 immunostaining; Student’s *t* test for plaque size and plaque density). Scale bar: 100 μm.


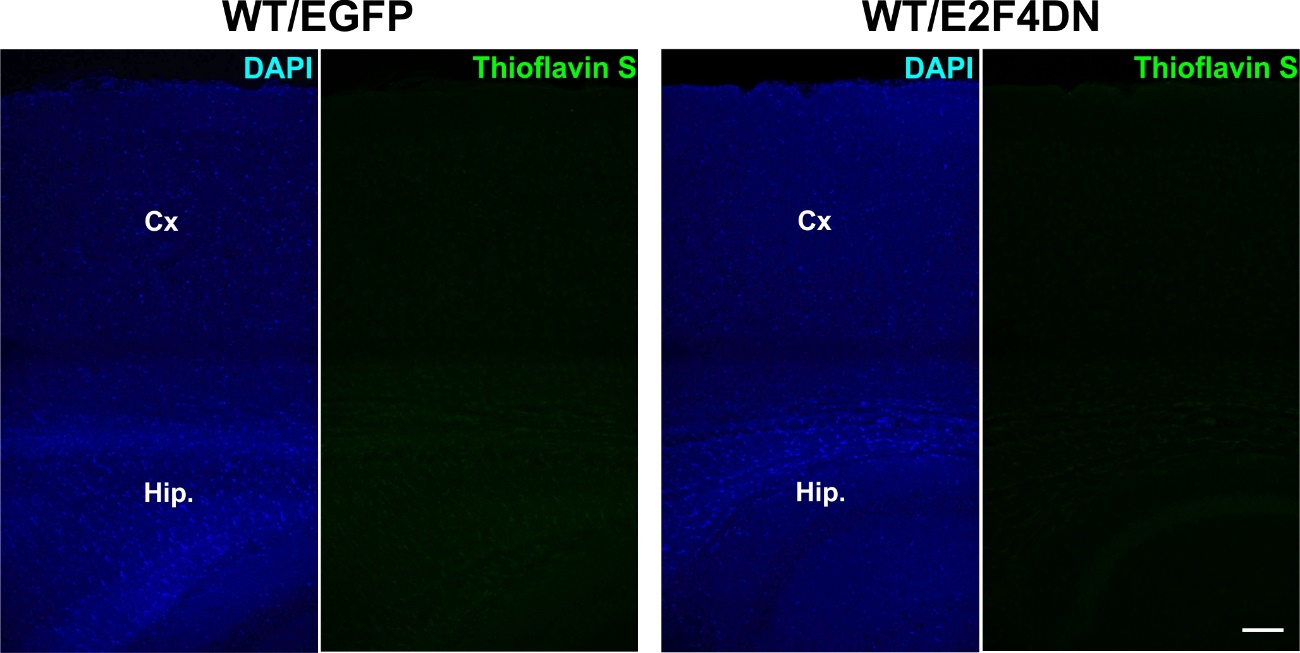


**Supplementary Fig. 5** Absence of dense core plaques in the brain of WT mice. Thioflavin S does not stain the cerebral cortex (Cx) of 6 month-old WT mice expressing either EGFP or E2F4DN. Scale bar: 100 μm.


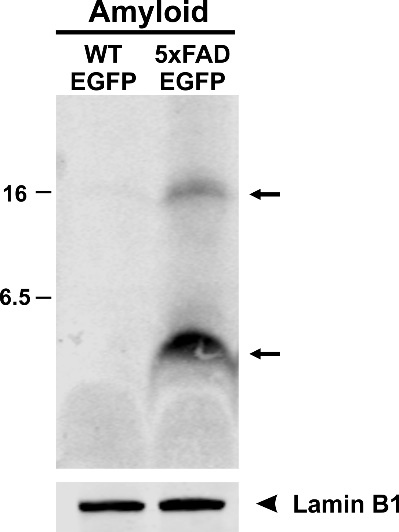


**Supplementary Fig. 6** Western blot analysis of hippocampal extracts from 5 month-old mice from the indicated genotypes using antibodies against Aβ and Lamin B1 (as a loading control). Arrows point to the monomer and tetramer forms of Aβ.


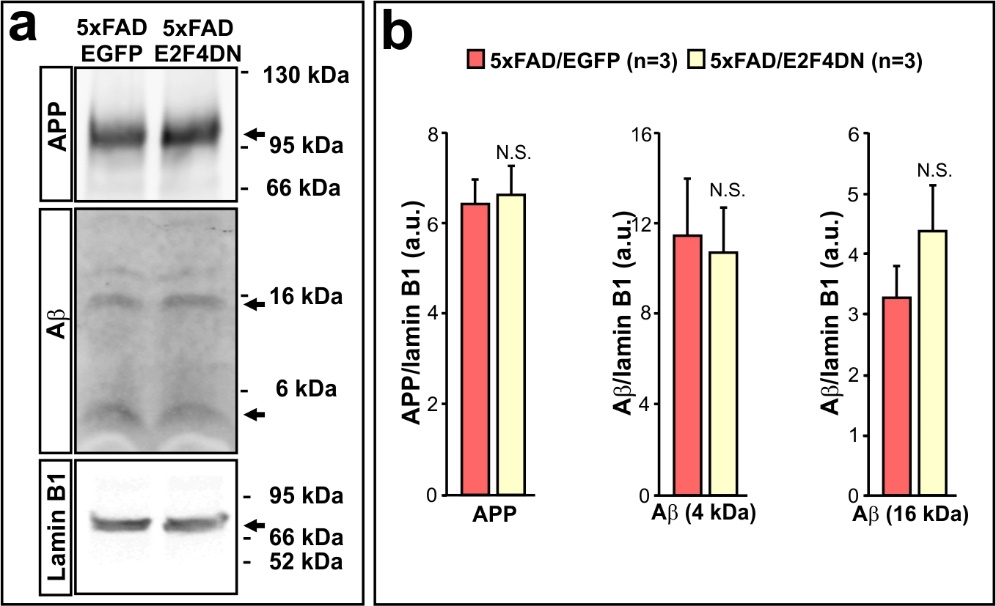


**Supplementary Fig. 7** Aβ production is not affected in 5xFAD mice with neuronal expression of E2F4DN. **a** Western blot analysis of hippocampal extracts from 5 month-old mice from the indicated genotypes using antibodies against APP, Aβ, and Lamin B1 (as a loading control). **b** Quantification of the western blots illustrated in A. Ratios against Lamin B1 are shown. N.S.: non-significant (Student’s *t* test). 5xFAD/EGFP: 3 females,

5xFAD/E2F4DN: 3 females.


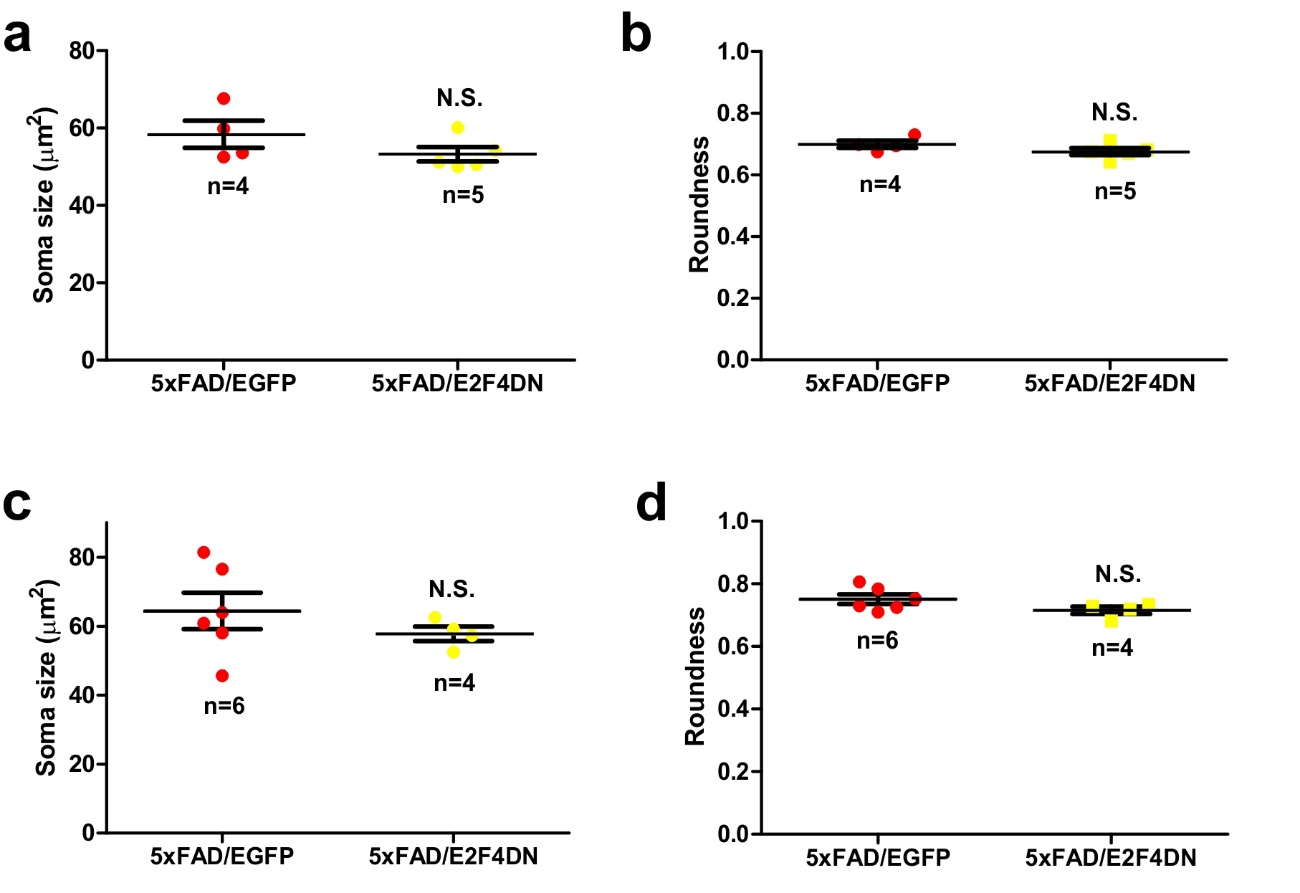


**Supplementary Fig. 8** Soma size and roundness of Iba1-positive microglial cells in the cerebral cortex of 5xFAD mice. **a, b** Analysis performed in the cerebral cortex of the indicated genotypes at 3 months of age: 5xFAD/EGFP (3 males and 1 female) and 5xFAD/E2F4DN (4 males and 1 female). **c, d** Analysis performed in the cerebral cortex of the indicated genotypes at 6 months of age: 5xFAD/EGFP (3 males and 3 females) and 5xFAD/E2F4DN (4 males). N.S.: non-significant (Student’s t test).


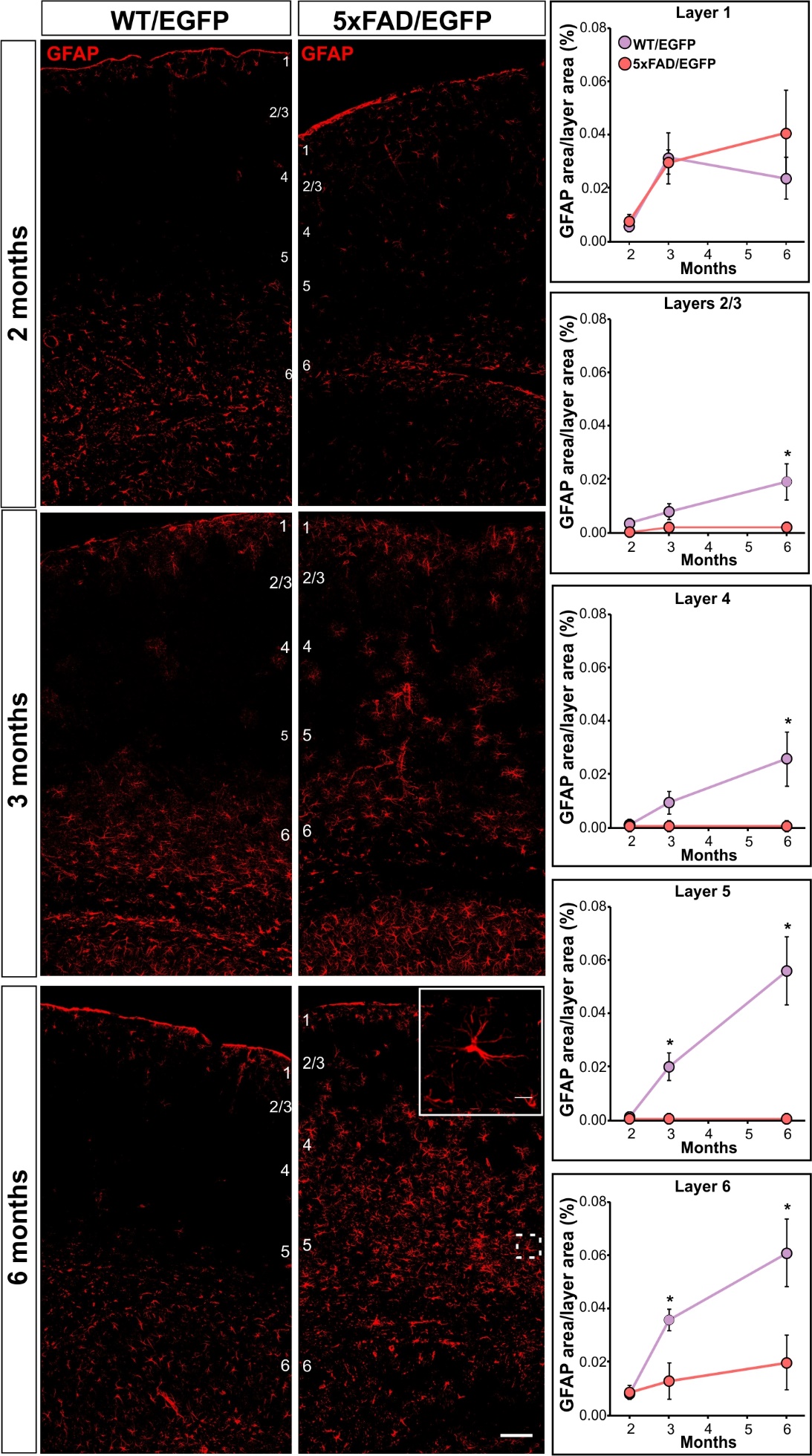


**Supplementary Fig. 9** Temporal course of GFAP-specific immunostaining in the cerebral cortex of mice of the indicated genotypes. Left panels: representative images of the expression pattern of GFAP at the indicated time points. Right panels illustrate the quantification of the percentage of area occupied by GFAP at the different temporal points. Inserts show high magnifications of the indicated dashed boxes. WT/EGFP: 2 months (1 male and 2 females), 3 months (1 male and 4 females), 6 months (4 males and 2 females); 5xFAD/EGFP: 2 months (2 males and 1 female), 3 months (4 males and 1 female), 6 months (3 males and 3 females); *p<0.05 (two-way ANOVA). Scale bar: 100 μm.


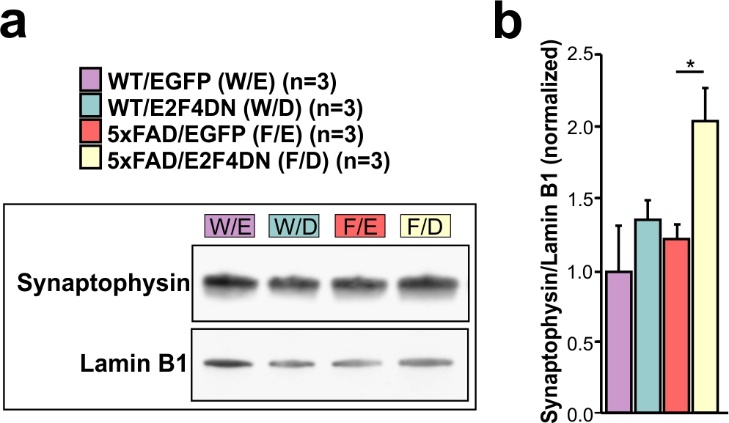


**Supplementary Fig. 10** Upregulation of synaptophysin in 5xFAD mice with neuronal expression of E2F4DN. **a** Western blot analysis of hippocampal extracts from 3 month-old mice from the indicated genotypes using antibodies against Synaptophysin and Lamin B1 (as a loading control). **b** Quantification of the western blots illustrated in a. Ratios against Lamin B1 are shown. *p<0.05 (Student’s *t* test). WT/EGFP: 3 females, WT/E2F4DN: 1 male and 2 females, 5xFAD/EGFP: 3 females, 5xFAD/E2F4DN: 3 females.


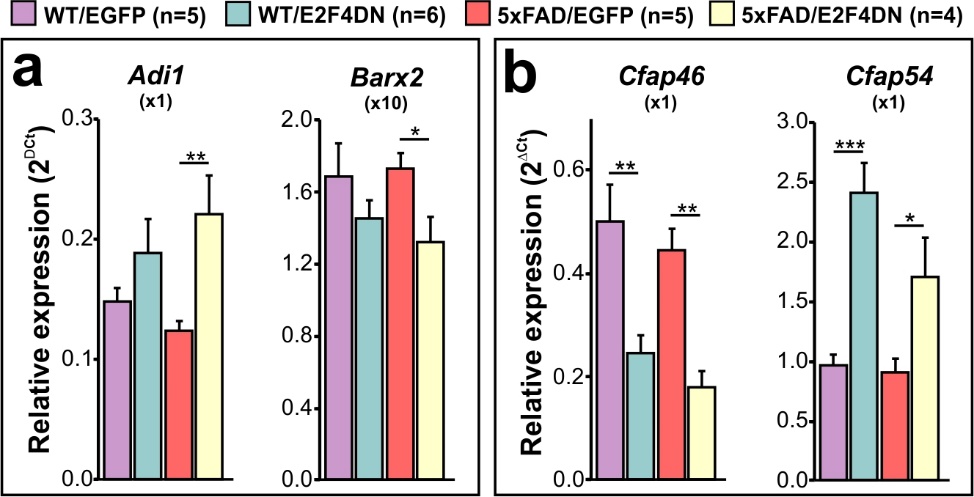


**Supplementary Fig. 11** Gene expression analysis by qPCR of E2F4-regulated genes of unclear connection with Alzheimer. **a, b** Genes whose expression is modulated by E2F4DN in the hippocampus of 3 month-old mice of the indicated genotypes are shown. Relative gene expression was normalized to *Rps18* rRNA levels and expressed as 2ΔCt (obtained values were adjusted by the factor indicated between brackets). *p<0.05; **p<0.01; ***p<0.001 (Unbalanced two-way ANOVA, followed by Student’s *t* test). WT/EGFP (5 females), WT/E2F4DN (3 males and 3 females), 5xFAD/EGFP (5 females), and 5xFAD/E2F4DN (4 females).


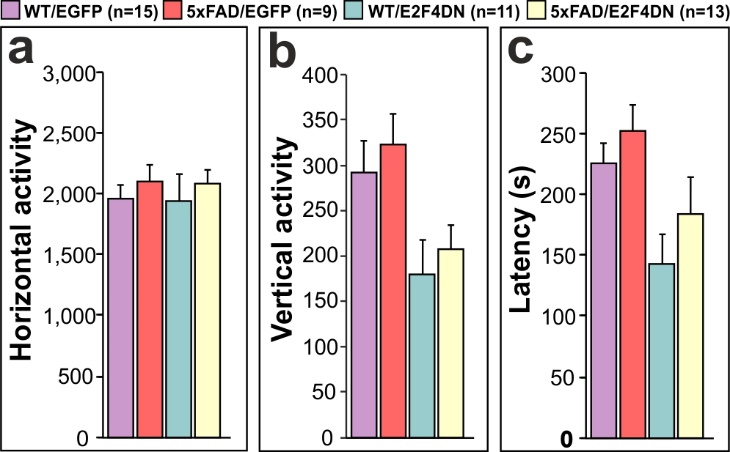


**Supplementary Fig. 12** Exploratory locomotor activity and motor coordination in 6-month-old male mice of the indicated genotypes. **a** Average number of infrared beams broken per trial over a 10 min period due to horizontal activity from crosses between heterozygous 5xFAD transgenic mice and either homozygous EGFP mice (left) or homozygous E2F4DN mice (right). **b** Average number of infrared beams broken per trial over a 10 min period due to vertical activity from crosses between heterozygous 5xFAD transgenic mice and either homozygous EGFP mice (left) or homozygous E2F4DN mice (right). **c** Motor coordination as measured by the rotarod text in 6-month-old littermates from crosses between heterozygous 5xFAD transgenic mice and either homozygous EGFP mice (left) or homozygous E2F4DN mice (right), measured as the time spent in the rotating rod before falling (latency). N.S.: non-significant (Unbalanced two-way ANOVA, followed by Student’s *t* test).

**
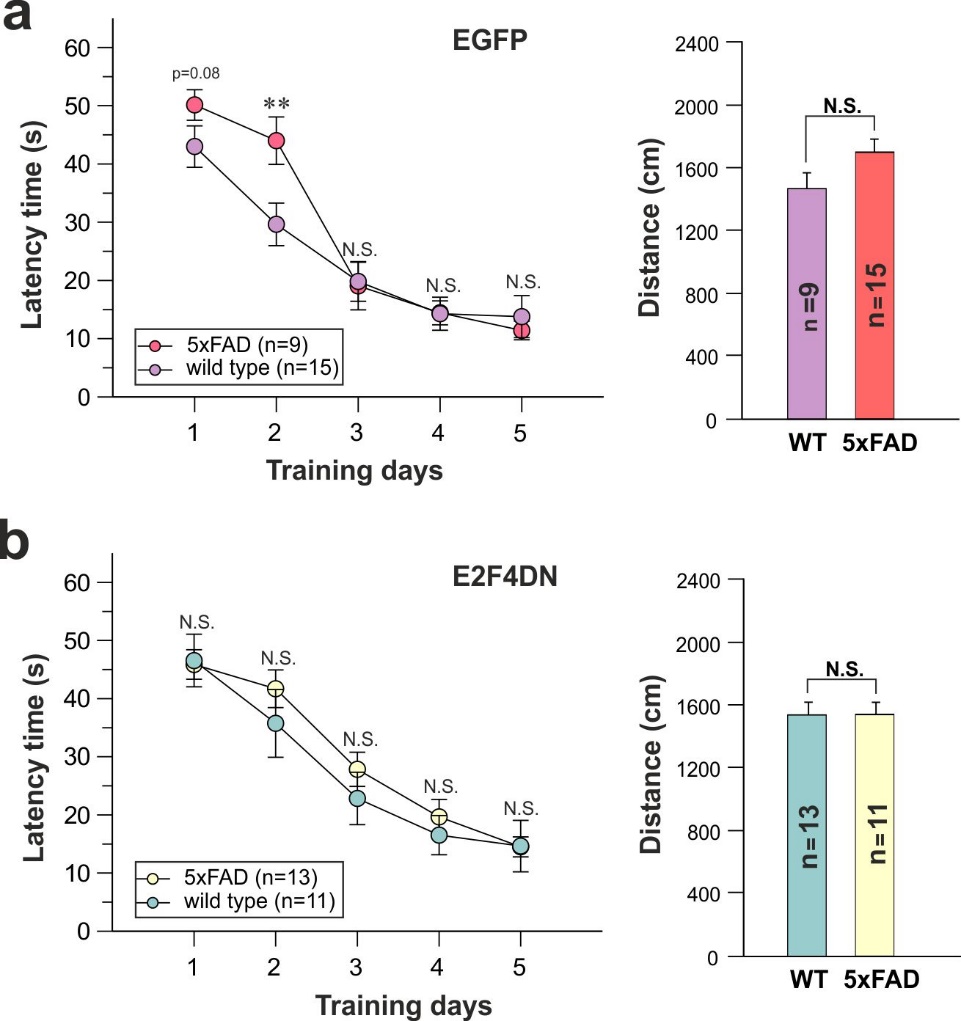
**

**Supplementary Fig. 13** mE2F4DN-myc expression prevents spatial memory impairment in 5xFAD mice as evaluated by the MWM test. **a** Latency time to reach the hidden platform along five training days in six-month-old littermates from crosses between heterozygous 5xFAD transgenic mice and homozygous EGFP mice. Both distance travelled and velocity was similar in both genotypes. **b** Latency time to reach the hidden platform along five training days in six five-month-old littermates from crosses between heterozygous 5xFAD transgenic mice and homozygous mE2F4DN-myc mice. Both distance travelled and velocity was similar in both genotypes. **p<0.01 (Student’s t test).


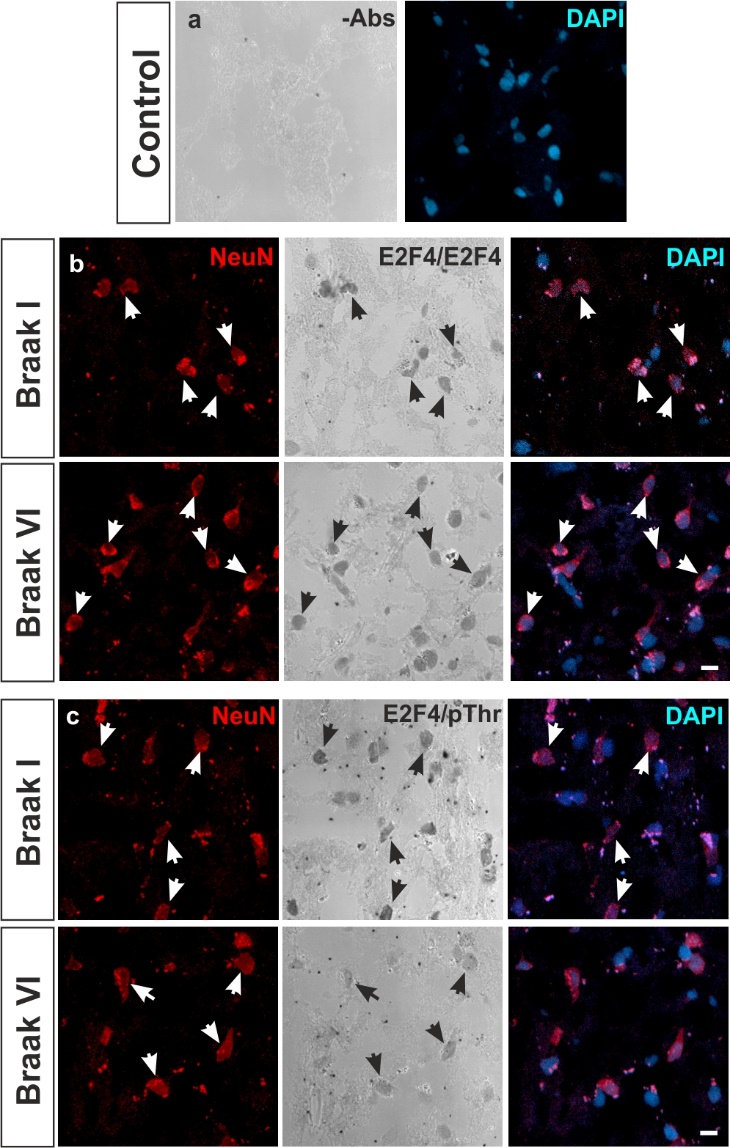


**Supplementary Fig. 14** Expression of E2F4 in the AD-affected parietal cortex. Parietal cortex cryosections from AD-affected individuals at Braak stages I and VI were subjected to PLA labeling (grey) in the absence of primary antibodies (a) or in the presence of a rabbit antibody against E2F4 together with a mouse antibody against either E2F4 (b) or phosphoThr (c). A chicken anti-NeuN antibody was used to identify neurons (red), and cell nuclei were revealed with DAPI staining (blue). Arrows: NeuN-positive cells. Scale bar: 5 μm.
