# Supplementary material for: A Mutant Variant of E2F4 Triggers Multifactorial Therapeutic Effects in 5xFAD Mice
Source: Mol Neurobiol. 2022 Mar 7;59(5):3016–39. doi: 10.1007/s12035-022-02764-z (PMC9016056; doi:10.1007/s12035-022-02764-z)
Supplement: Supplementary file 2 — (pptx 1.55 MB) [file 12035_2022_2764_MOESM2_ESM.pptx]

## Slide 1
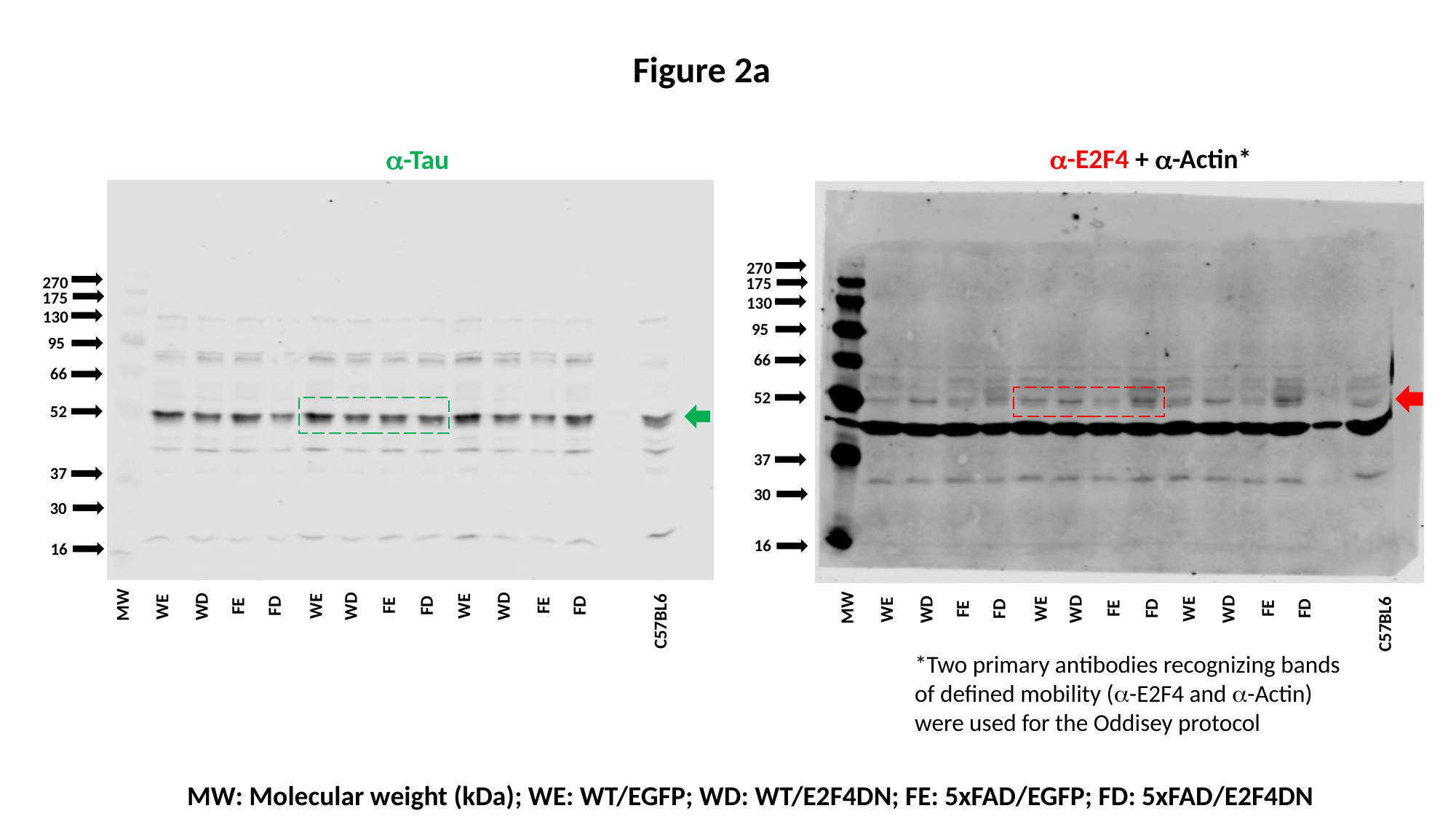

Figure 2a
a-E2F4 + a-Actin*
a-Tau
270
175
130
95
66
52
37
30
16
270
175
130
95
66
52
37
30
16
MW
FE
FD
FE
FD
FE
FD
WE
WD
WE
WD
WE
WD
C57BL6
MW
FE
FD
FE
FD
FE
FD
WE
WD
WE
WD
WE
WD
C57BL6
*Two primary antibodies recognizing bands of defined mobility (a-E2F4 and a-Actin) were used for the Oddisey protocol
MW: Molecular weight (kDa); WE: WT/EGFP; WD: WT/E2F4DN; FE: 5xFAD/EGFP; FD: 5xFAD/E2F4DN

## Slide 2
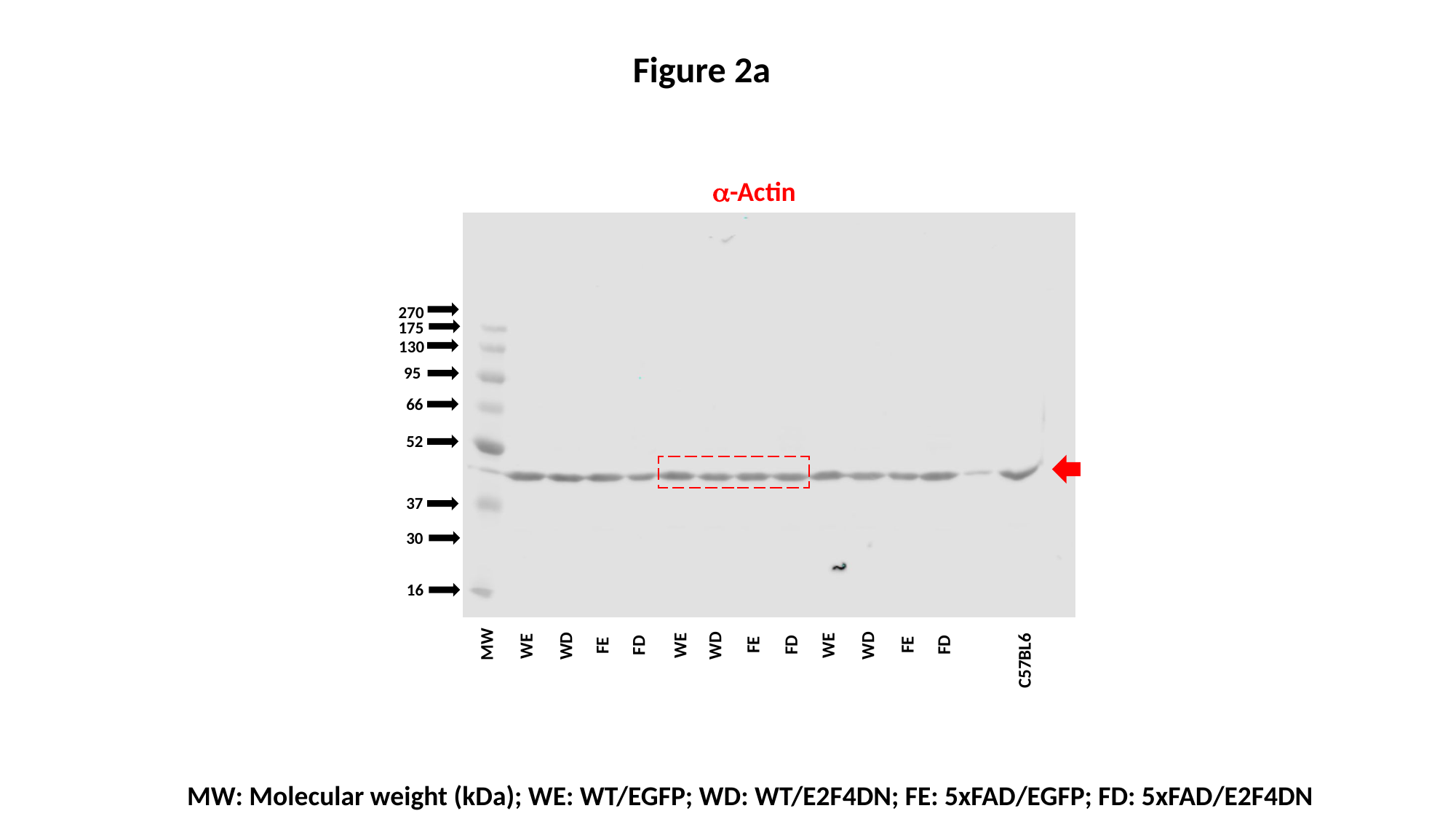

Figure 2a
a-Actin
270
175
130
95
66
52
37
30
16
MW
FE
FD
FE
FD
FE
FD
WE
WD
WE
WD
WE
WD
C57BL6
MW: Molecular weight (kDa); WE: WT/EGFP; WD: WT/E2F4DN; FE: 5xFAD/EGFP; FD: 5xFAD/E2F4DN

## Slide 3
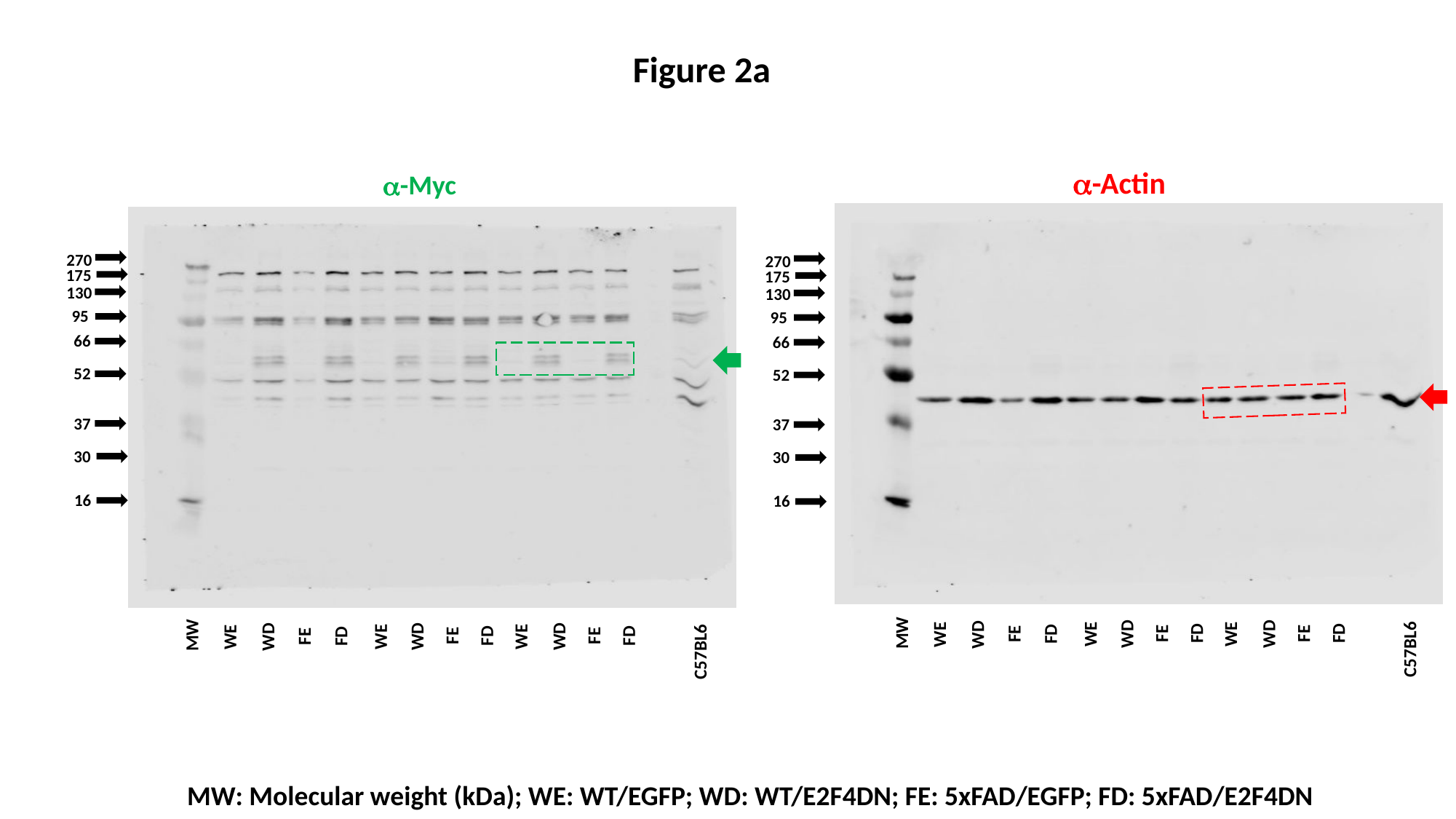

Figure 2a
a-Actin
a-Myc
270
175
130
95
66
52
37
30
16
270
175
130
95
66
52
37
30
16
MW
FE
FD
FE
FD
FE
FD
WE
WD
WE
WD
WE
WD
C57BL6
MW
FE
FD
FE
FD
FE
FD
WE
WD
WE
WD
WE
WD
C57BL6
MW: Molecular weight (kDa); WE: WT/EGFP; WD: WT/E2F4DN; FE: 5xFAD/EGFP; FD: 5xFAD/E2F4DN

## Slide 4
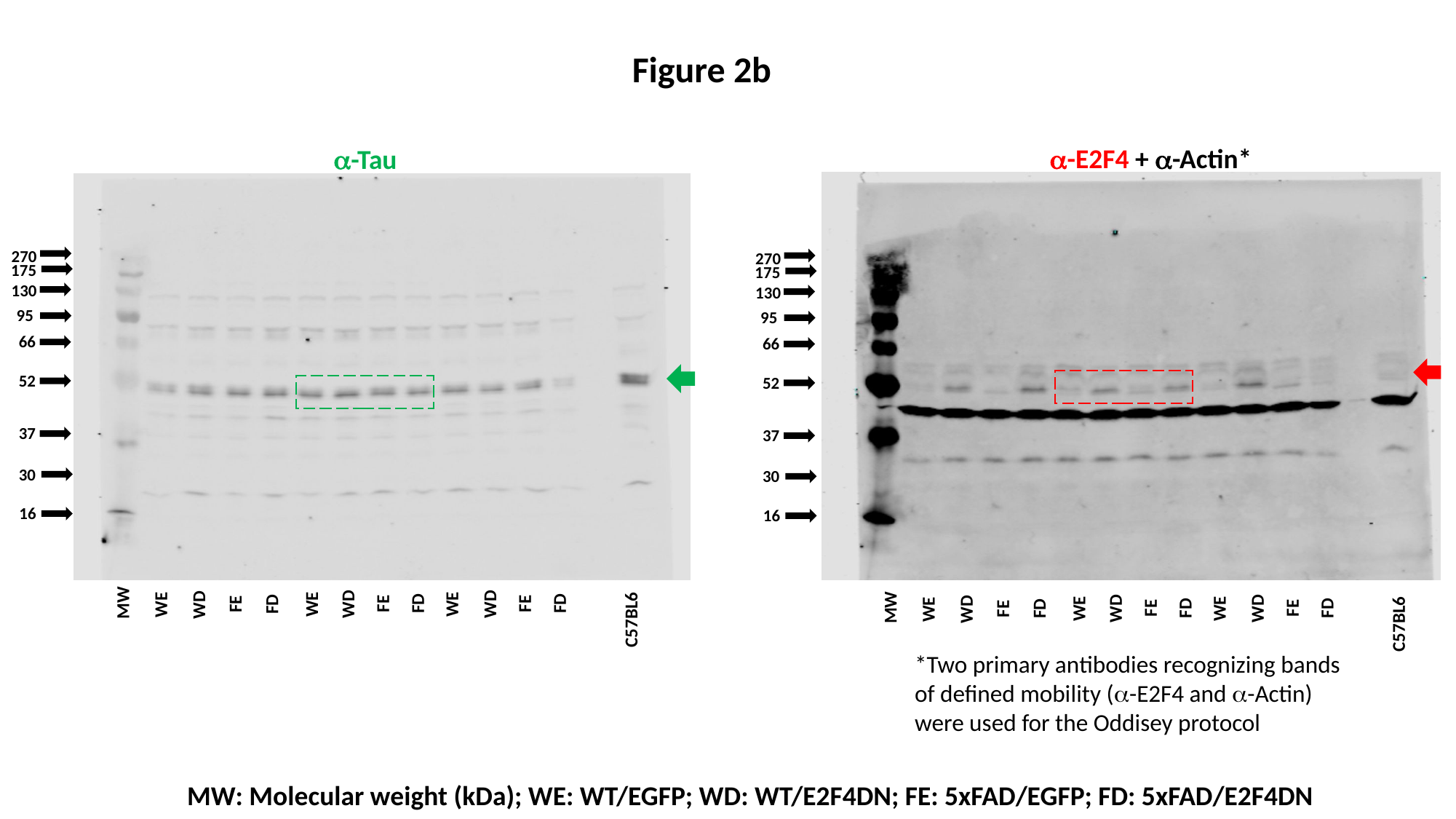

Figure 2b
a-E2F4 + a-Actin*
a-Tau
270
175
130
95
66
52
37
30
16
270
175
130
95
66
52
37
30
16
MW
FE
FD
FE
FD
FE
FD
WE
WD
WE
WD
WE
WD
C57BL6
MW
FE
FD
FE
FD
FE
FD
WE
WD
WE
WD
WE
WD
C57BL6
*Two primary antibodies recognizing bands of defined mobility (a-E2F4 and a-Actin) were used for the Oddisey protocol
MW: Molecular weight (kDa); WE: WT/EGFP; WD: WT/E2F4DN; FE: 5xFAD/EGFP; FD: 5xFAD/E2F4DN

## Slide 5
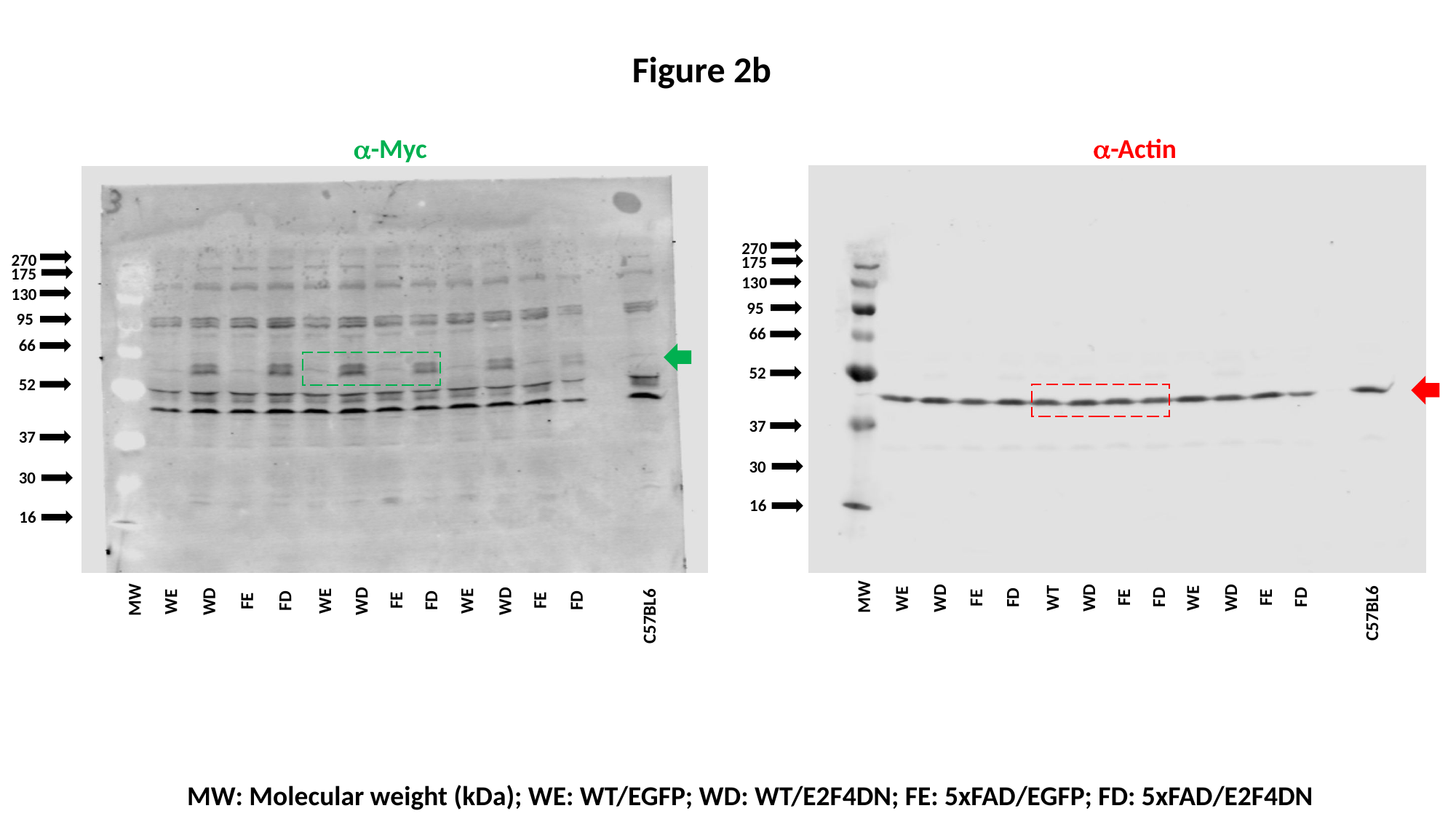

Figure 2b
a-Actin
a-Myc
270
175
130
95
66
52
37
30
16
270
175
130
95
66
52
37
30
16
MW
FE
FD
FE
FD
FE
FD
WT
WD
WE
WD
WE
WD
C57BL6
MW
FE
FD
FE
FD
FE
FD
WE
WD
WE
WD
WE
WD
C57BL6
MW: Molecular weight (kDa); WE: WT/EGFP; WD: WT/E2F4DN; FE: 5xFAD/EGFP; FD: 5xFAD/E2F4DN

## Slide 6
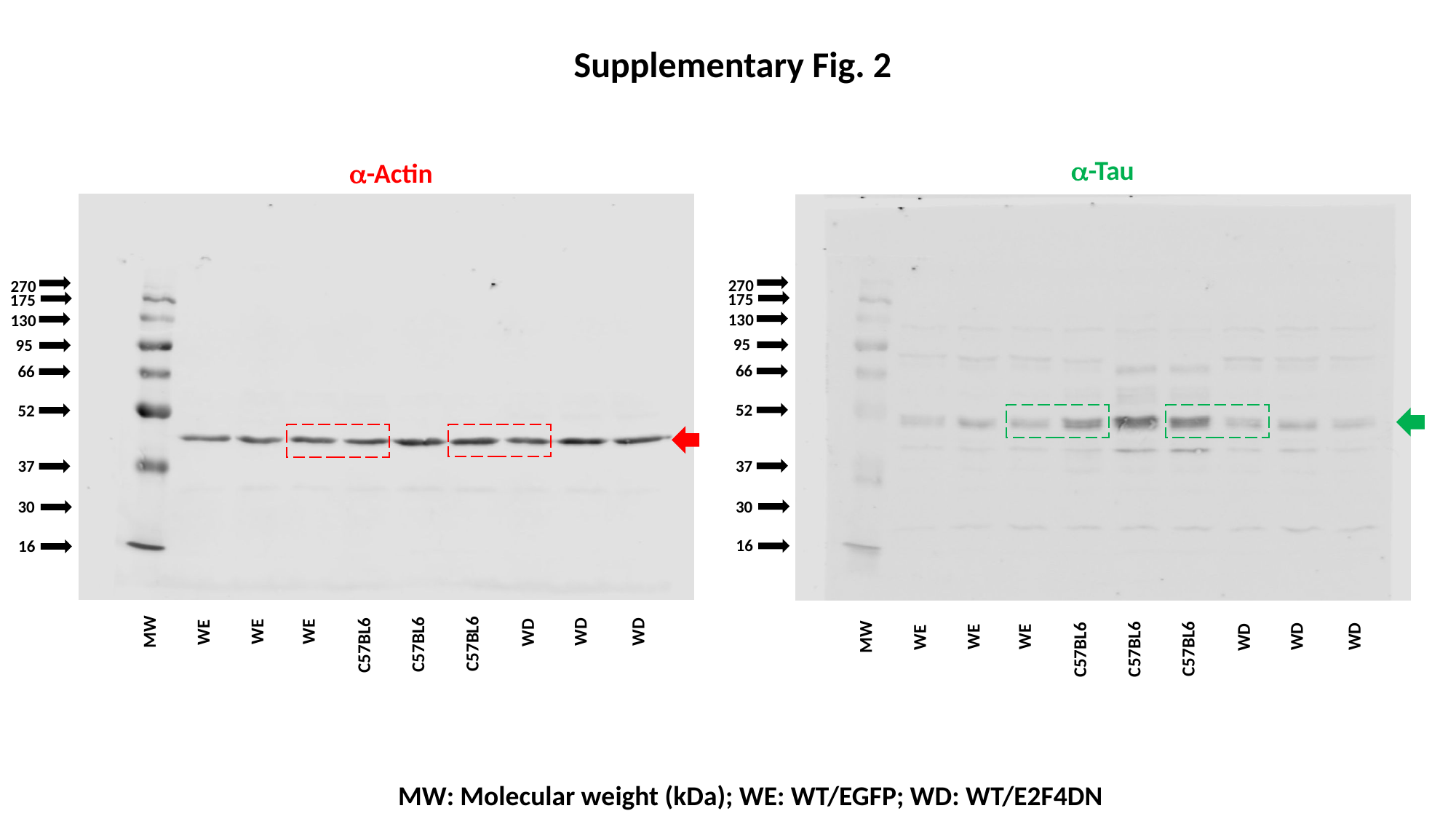

Supplementary Fig. 2
a-Tau
a-Actin
270
175
130
95
66
52
37
30
16
270
175
130
95
66
52
37
30
16
MW
WE
WE
WD
WD
WE
WD
C57BL6
C57BL6
C57BL6
MW
WE
WE
WD
WD
WE
WD
C57BL6
C57BL6
C57BL6
MW: Molecular weight (kDa); WE: WT/EGFP; WD: WT/E2F4DN

## Slide 7
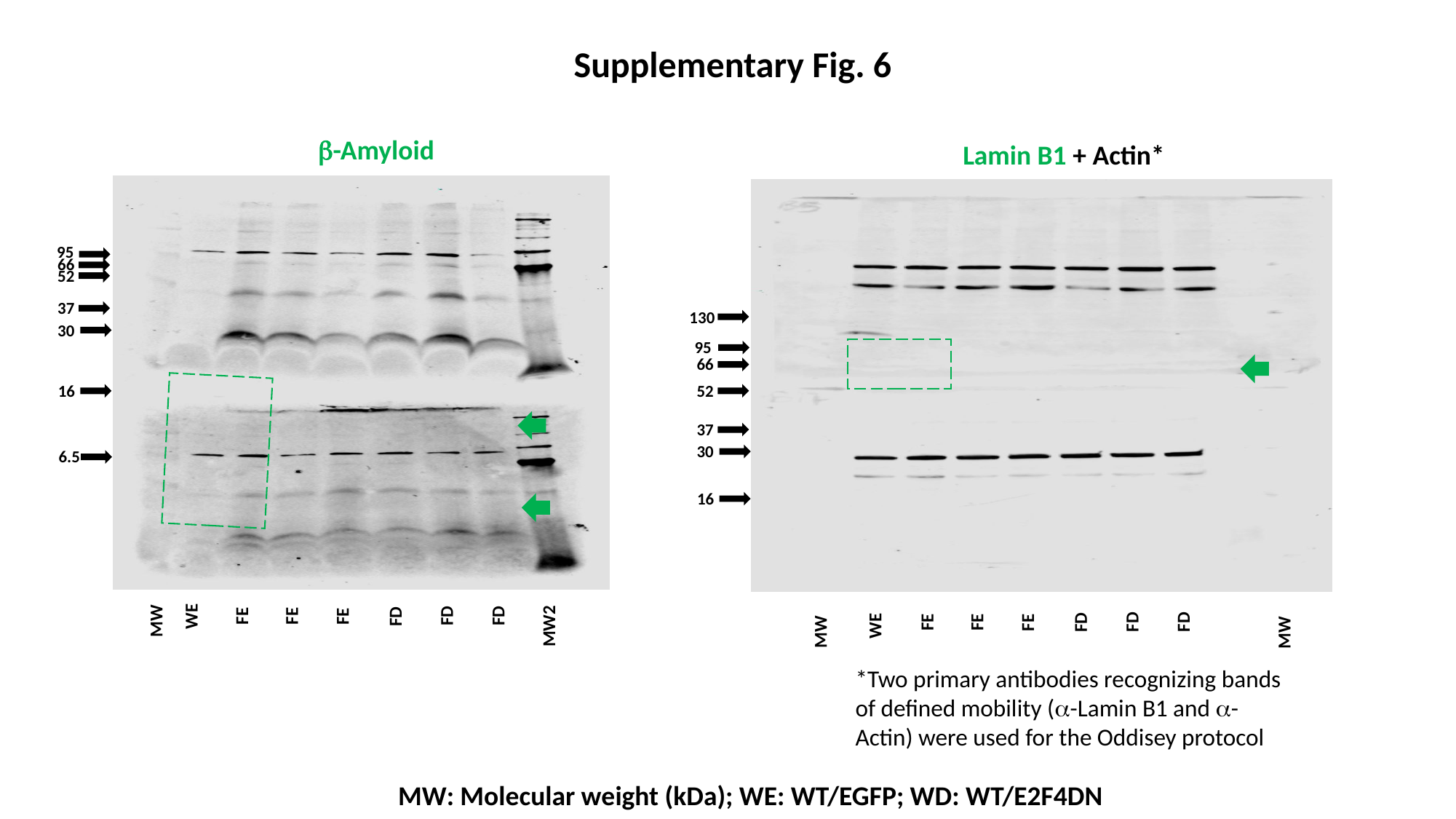

Supplementary Fig. 6
b-Amyloid
Lamin B1 + Actin*
95
66
52
37
30
16
6.5
130
95
66
52
37
30
16
FE
FE
FD
FD
WE
FD
FE
MW
MW2
FE
FE
FD
FD
FD
FE
WE
MW
MW
*Two primary antibodies recognizing bands of defined mobility (a-Lamin B1 and a-Actin) were used for the Oddisey protocol
MW: Molecular weight (kDa); WE: WT/EGFP; WD: WT/E2F4DN

## Slide 8
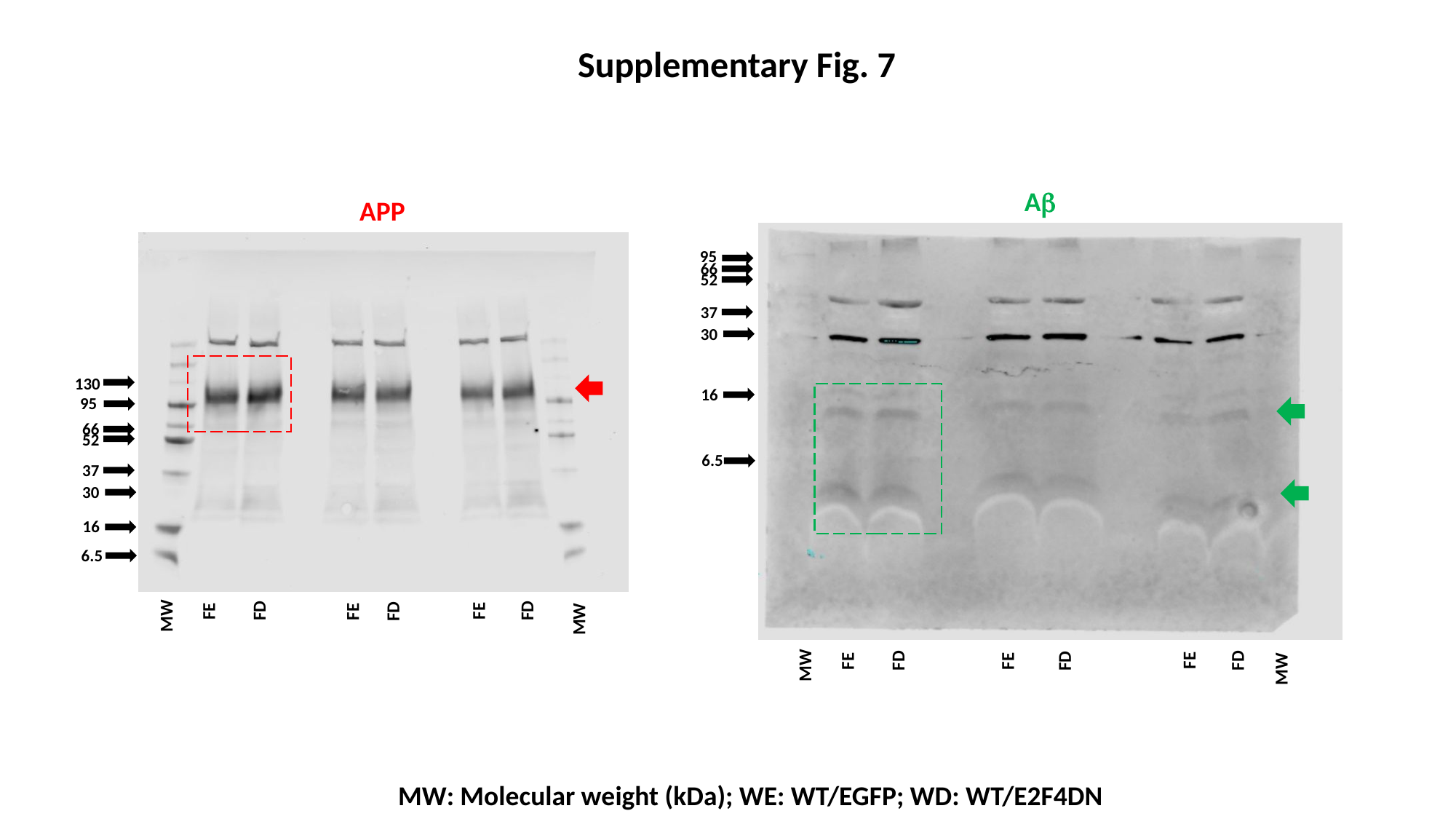

Supplementary Fig. 7
Ab
APP
95
66
52
37
30
16
6.5
130
95
66
52
37
30
16
6.5
FD
FE
FD
FE
FD
FE
MW
MW
FD
FE
FD
FE
FD
FE
MW
MW
MW: Molecular weight (kDa); WE: WT/EGFP; WD: WT/E2F4DN

## Slide 9
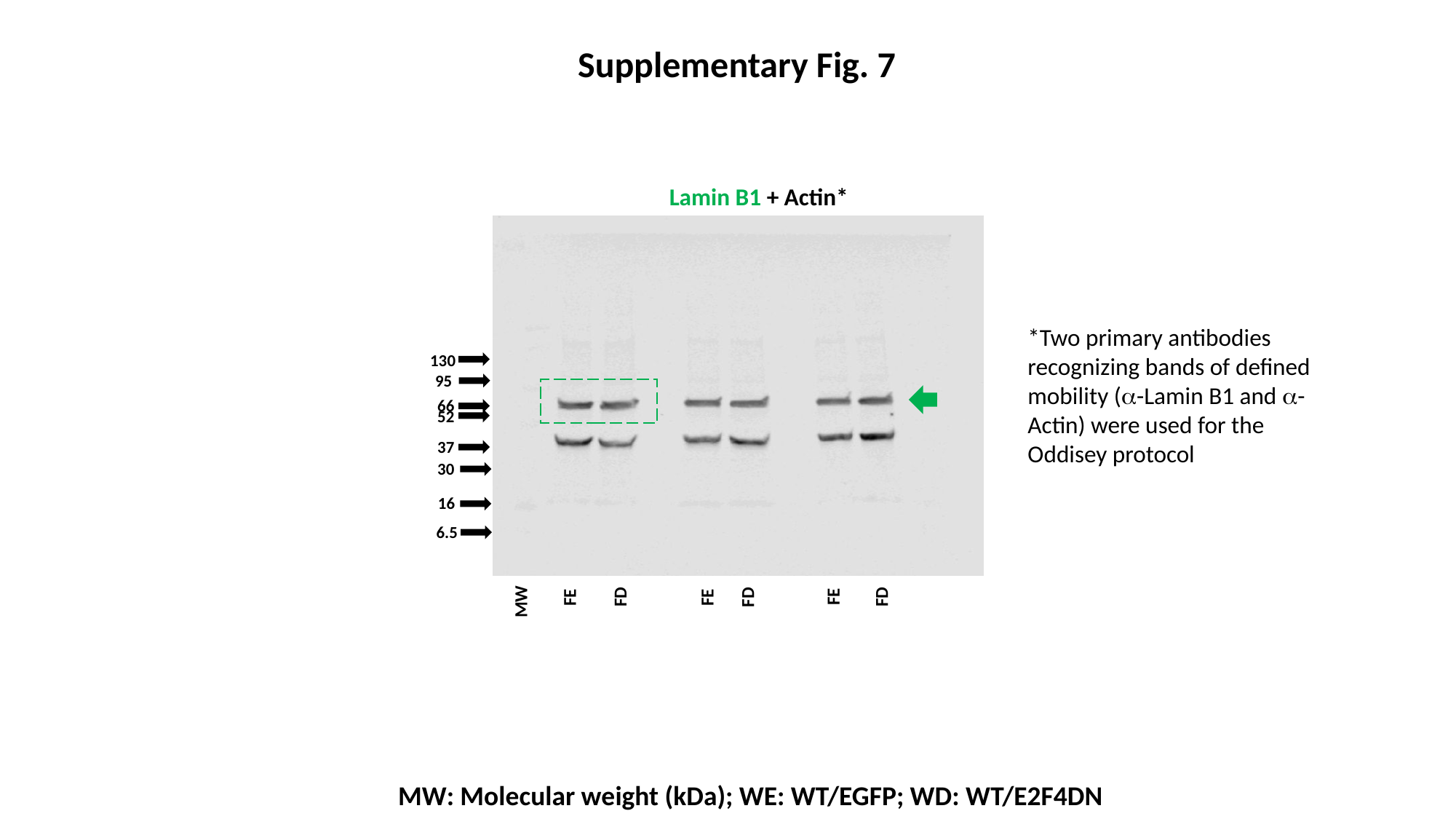

Supplementary Fig. 7
Lamin B1 + Actin*
*Two primary antibodies recognizing bands of defined mobility (a-Lamin B1 and a-Actin) were used for the Oddisey protocol
130
95
66
52
37
30
16
6.5
FD
FE
FD
FE
FD
FE
MW
MW: Molecular weight (kDa); WE: WT/EGFP; WD: WT/E2F4DN

## Slide 10
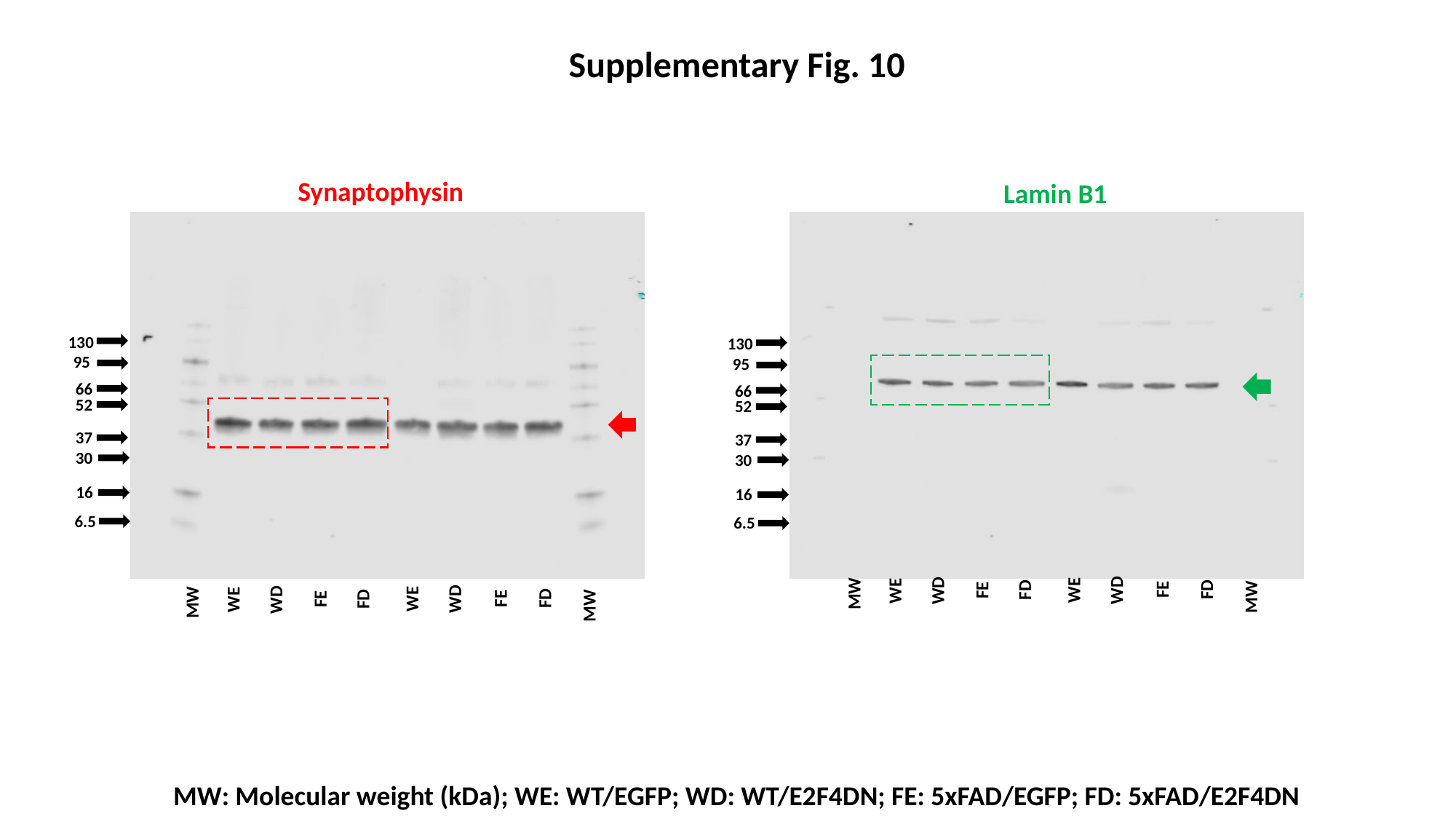

Supplementary Fig. 10
Synaptophysin
Lamin B1
130
95
66
52
37
30
16
6.5
130
95
66
52
37
30
16
6.5
FE
FD
FE
FD
WE
WD
WE
WD
MW
MW
FE
FD
FE
FD
WE
WD
WE
WD
MW
MW
MW: Molecular weight (kDa); WE: WT/EGFP; WD: WT/E2F4DN; FE: 5xFAD/EGFP; FD: 5xFAD/E2F4DN
